# Testosterone Delays Bone Microstructural Destruction via Osteoblast‐Androgen Receptor‐Mediated Upregulation of Tenascin‐C

**DOI:** 10.1002/advs.202501518

**Published:** 2025-05-30

**Authors:** Yong Xie, Meng Pan, Zeyuan Zhang, Licheng Zhang, Haotian Liu, Xia Wang, William W Lu, Peifu Tang, Wei Ge

**Affiliations:** ^1^ Department of Orthopedics The Fourth Medical Center Chinese PLA General Hospital Beijing 100048 China; ^2^ State Key Laboratory of Complex, Severe, and Rare Diseases Department of Immunology Institute of Basic Medical Sciences Chinese Academy of Medical Sciences School of Basic Medicine Peking Union Medical College Beijing 100005 China; ^3^ National Clinical Research Center for Orthopedics Sports Medicine & Rehabilitation Beijing 100048 China; ^4^ Department of Orthopaedics and Traumatology The University of Hong Kong Hong Kong 999077 China

**Keywords:** bone destruction, male bones, osteoblastic androgen receptor, tenascin‐C

## Abstract

Bone loss and microstructural destruction in elderly men are associated with fractures and high mortality. While testosterone (Tes) is considered to be possibly protective, its regulatory mechanism in bone remodeling remains unclear. Here, bone microarchitectural analysis indicates that elderly men exhibit reduced cortical and trabecular thickness with elevated cortical porosity, particularly at the superior femoral head near the medial acetabulum. Serum profiling of 352 individuals showed that low Tes levels (<9.415 nmol·L^−1^) are associated with higher risk of bone loss. In vivo, tail‐suspended mice lacking osteoblastic androgen receptor (AR) displayed similar femoral deterioration, with decreased trabecular bone and increased cortical porosity. Mechanistically, Tes enhances osteoblastic differentiation via AR‐mediated upregulation of tenascin‐C (TNC). Molecular docking suggests the fibrinogen C‐terminal domain of TNC inhibits osteoclastogenesis by binding integrin αV, blocking adhesion of RGD‐containing proteins. A synthetic peptide (pep2) mimicking this domain preserved bone architecture in osteoblast‐specific *Ar*‐knockout, tail‐suspended mice. Moreover, elevated serum extracellular vesicle amyloid precursor protein, secondary to Tes‐AR‐TNC decline and osteoclast overactivation, emerged as a biomarker of bone loss when combined with low Tes. This study identifies the Tes‐AR‐TNC axis as a key regulator of male bone remodeling, offering insights into fracture risk assessment and targeted interventions in bone destruction.

## Graphical abstract

This study reveals that Testosterone–Androgen Receptor signaling delays elderly male bone destruction by upregulation of the osteoblastic extracellular tenascin‐C (TNC). The osteoprotective effect of fibrinogen C‐terminus of TNC is demonstrated in male osteoporotic mice model that osteoblast‐specific *Ar*‐knockout, potentially via inhibition of osteoclastogenesis by binding with integrin αV, which blocks the adhesion of pro‐osteoclastic proteins with RGD motif.

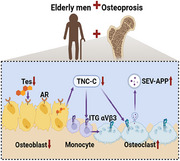

## 1 Introduction

Bone loss in elderly males is associated with elevated mortality due to fractures, although the overall prevalence of major fragility fractures in older men is approximately one‐third that in women.^[^ ^1^, ^2^ ^]^ The cumulative mortality rate 1 year after hip fracture among men is as high as 20–40%, indicating that men are about twice as likely to die in hospital after a hip osteoporotic fracture as women, even after considering co‐morbidities, medications, and background mortality.^[^ ^3^, ^4^, ^5^, ^6^ ^]^ This is difficult to explain from the perspective of differences in the maintenance of skeletal homeostasis by sex hormones alone. In contrast to women, the majority of men do not typically experience overt hypogonadism as they age. It has been believed that androgen deficiency is not a significant factor in age‐related male diseases.^[^ ^7^ ^]^ However, a growing body of evidence indicates that low androgen levels are associated with bone loss in elderly men,^[^ ^8^ ^]^ suggesting that the regulatory role of androgen in aged male bones deserves more attention, and it is not enough to directly extrapolate to males what is known for females. Recent analysis with high‐resolution peripheral quantitative computed tomography demonstrated that bone microarchitecture was not compromised in trans‐men who received testosterone (Tes, a primary androgen) therapy and suppression of ovarian estradiol production, whereas trans‐women receiving 17‐β estradiol (β‐E2), with or without antiandrogen therapy, had deteriorated bone microarchitecture, implying that effects of estradiol were insufficient to counteract the reduced capacity of Tes to maintain bone strength,^[^ ^9^ ^]^ which emphasizes the pivotal role of Tes in protecting bone quality in men.^[^ ^10^ ^]^ Therefore, elucidating specific Tes‐mediated bone remodeling mechanisms in males is essential to develop precision interventions for the deterioration of bone mass and structure in elderly men, rather than relying solely on using general Tes replacement therapy (TRT).

As the major testicular factor and best‐known androgen,^[^ ^11^, ^12^ ^]^ Tes mediates signaling cascades by binding to its classical nuclear hormone receptor, androgen receptor (AR),^[^ ^13^ ^]^ which is widely expressed in bone cells, including osteoclasts,^[^ ^14^ ^]^ osteoblasts and their precursors.^[^ ^15^, ^16^ ^]^ Following orchiectomy (ORX) in mice, the rise in the RANKL/OPG mRNA ratio in bone tissue is counteracted by mechanical loading, suggesting that physical activity could mitigate bone loss in conditions of androgen deficiency by exerting an antiresorptive influence.^[^ ^17^ ^]^ While mice with specific deletion of AR in osteoclasts showing that androgen signaling within osteoclasts does not contribute to the bone‐preserving effects of androgens in trabecular or cortical bone compartments.^[^ ^18^ ^]^ By contrast, mice with specific deletion of AR in osteoblasts exhibited decreased trabecular volume and trabecular number (Tb.N) associated with an increase in osteoclast number; however, no effects on cortical bone mass and turnover were demonstrated. This evidence is consistent with clinical findings that the maintenance of Tes is associated with the protection of trabecular bone morphology and biomechanical properties.^[^ ^19^, ^20^ ^]^ Furthermore, the absence of changes in RANKL/OPG level due to AR deletion in osteoblasts and osteocytes implies that alternative mechanisms may account for the antiresorptive impact of the androgen‐AR signaling pathway on trabecular bone.^[^ ^21^ ^]^ Thus, we inferred that the Tes attenuates osteoclastogenesis in trabecular bone indirectly via osteoblasts‐AR, while the spatiotemporal characteristics of bone deterioration in elderly men and the underlying mechanisms of Tes in the regulation of bone remodeling have not yet been elucidated.

Among the four vertebrate tenascins, a role in skeletal function has been attributed to tenascin‐C (TNC), an extracellular matrix glycoprotein implicated in osteogenic differentiation and the process of bone mineralization.^[^ ^22^ ^]^ TNC typically adopts a characteristic hexameric structure, comprised of four distinct domains arranged from *N*‐terminus to *C*‐terminus as a coil‐like tenascin assembly domain, an epidermal growth factor‐like domain, a fibronectin type III (FN III)‐like domain, and a fibrinogen‐like globe (FBG) domain. This multimodular configuration facilitates the interaction of TNC with a broad array of diverse ligands, including annexin II, syndecan‐4, and integrins, via alternative splicing mechanisms. The precise regulation of distinct TNC cleavage is tightly controlled by the specific cell type and the microenvironment of the tissue,^[^ ^23^ ^]^ including biological factors and mechanical changes, each operating through unique signaling pathways, yet the reported control of TNC in bone remains poorly understood.^[^ ^24^ ^]^

In the present study, bone microarchitectural analysis indicated that age‐related bone destruction in the proximal femur of elderly males is mainly characterized by a decrease in the thickness of cortical and trabecular bone (with an increase in cortical porosity) in the superior portion of the femoral head close to the medial acetabulum. Serum detection from 352 individuals suggested that elderly men with low Tes (<9.415 nmol L^−1^) have a high risk of bone loss. In vivo studies using tail‐suspended mice lacking AR in osteoblasts demonstrated similar structural changes to that in elderly men, that is, bone destruction at the top of the proximal femur with decreased amount and thickness of trabecular bone, and increased cortical porosity and trabecular separation compared with AR*^flox/Y^* mice. Further in vitro study showed that Tes activates osteoblast‐AR‐mediated upregulation of the extracellular TNC during osteoblast differentiation, which indirectly inhibits osteoclastogenesis and bone resorption. Molecular docking analysis using ColabFold indicated that the underlying negative regulatory effect of the *C*‐terminus of TNC on osteoclastogenesis occurs via binding with integrin αV (ITGαV), thereby blocking the adhesion of pro‐osteoclastic protein with RGD motif, such as OPN, to osteoclasts. Additionally, excess serum extracellular vesicle amyloid‐beta precursor protein (SEV‐APP), resulting from osteoclastic overactivation following decreased Tes‐AR‐mediated expression of the fibronectin *C*‐terminus of TNC, was identified to be a potential biomarker for early risk assessment of bone loss in elderly men (when combined with low Tes). This study reveals that the Tes‐TNC‐APP axis delays bone destruction in elderly men and highlights the potential value in risk assessment of bone loss and nonunion, in addition to precision interventions for deterioration of bone mass and structure.

## 2 Results

### 2.1 Bone Loss and Microstructural Destruction in the Superior Portion of the Femoral Head in Elderly Males is Mainly Manifested by Decreases in the Thickness of Cortical and Trabecular Bone, Accompanied by a Low Serum Tes Level

**Figure 1:**
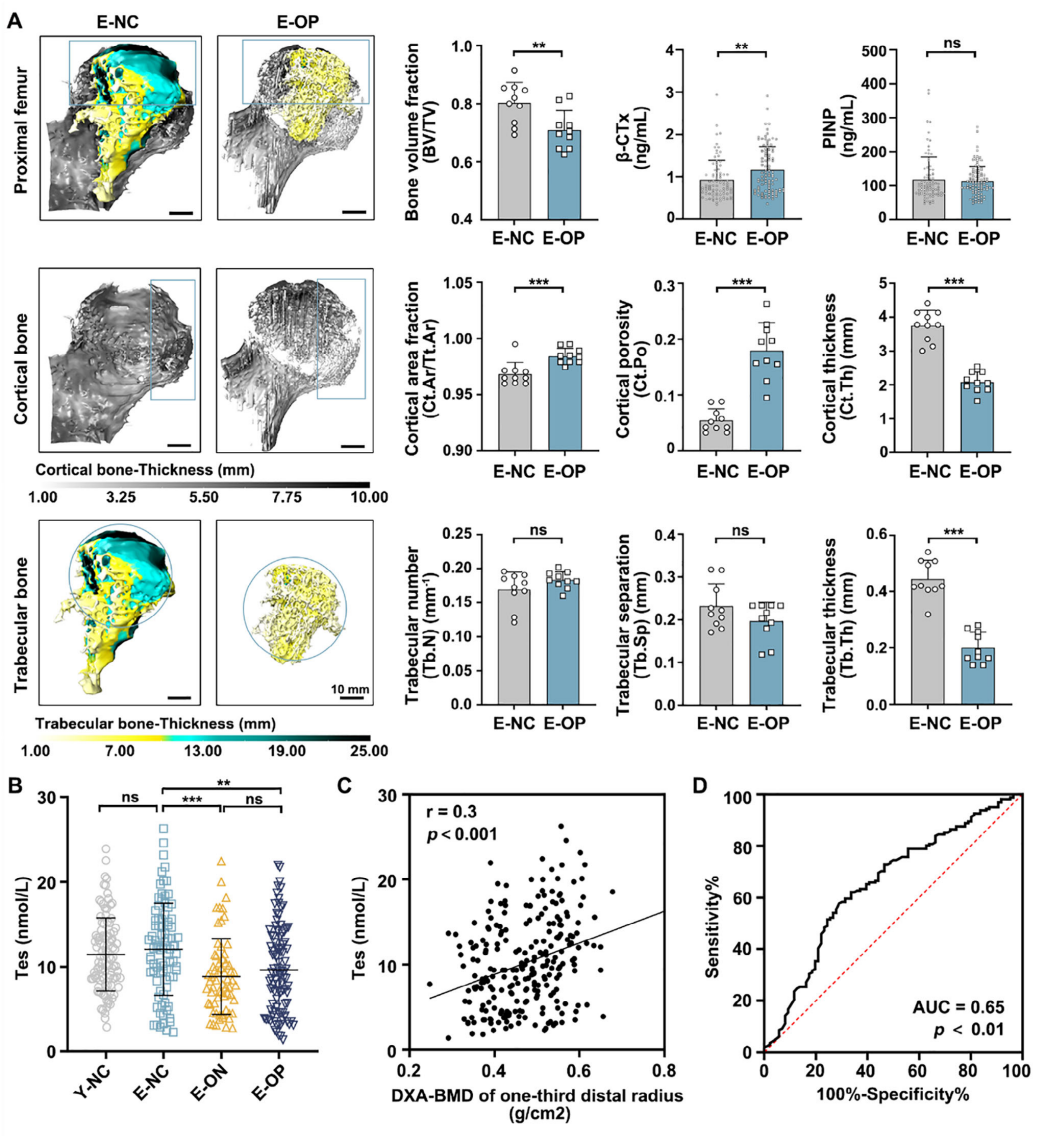
The thickness of cortical and trabecular bone in the superior portion of the femoral head decreases in elderly men with low serum Tes levels. A) 3D reconstruction and bone analysis of CT images of the right hip from elderly men, *n* = 10 per group; β‐CTx and PINP in serum of 97 elderly men with osteoporosis and 86 elderly men with normal BMD. B) Comparisons of Tes level in serum among young men with normal BMD (Y‐NC, *n* = 105), elderly men with normal BMD (E‐NC, *n* = 86), elderly men with osteopenia (E‐ON, *n* = 64), and elderly men with osteoporosis (E‐OP, *n *= 97). C) Spearman's correlation analysis between serum Tes level and DXA‐BMD of one‐third distal radius in elderly men (*n* = 247). (D) ROC curve analysis of serum Tes level (*n* = 86 for normal BMD; *n* = 161 for bone loss). Student's *t*‐test was used for two groups of comparisons, one‐way ANOVA with Tukey's multiple comparisons test was used for multiple comparisons. All tests were two‐sided; **p < 0.01; ***p < 0.001; ns = no significance. Tes = testosterone; DXA = dual‐energy X‐ray absorptiometry; BMD = bone mineral density; AUC = area under the curve.

To understand the spatiotemporal characteristics of bone degeneration in elderly men, computed tomography (CT) images of the right hip of 10 elderly men with normal bone mass and 10 elderly men with osteoporosis (OP) were included (both groups had equivalent baseline age and body size) (Figure S1A, Table S1, Supporting Information). The medial part of the trochanter and the area above the lesser trochanter of the proximal femur were determined as the volume of interest (VOI) using a standardized process as described in Methods. Compared with elderly men with normal bone mass, elderly men with OP showed a lower BV/TV in the proximal femur. For cortical bone, cortical thickness (Ct.Th) was significantly lower in the medial part of the femoral head of elderly men with OP, simultaneously appearing with higher cortical porosity (Ct.Po), representing the thinning and discontinuity of the cortical bone, which determines mechanical strength and resistance to external impact. For trabecular bone, a significantly lower trabecular thickness (Tb.Th) was observed at the top position of the femoral head for elderly men with OP compared with elderly men with normal bone mass (almost 2.5 times), while the trabecular number (Tb.N) and trabecular separation (Tb.Sp) showed no significant difference between elderly men with and without OP. Contrary to our hypothesis, the cortical area fraction (Ct.Ar/Tt.Ar) of elderly men with OP was slightly higher than those of elderly men with normal bone mass. Perhaps this result is related to our limited resolution, but it indicates that the bone loss and microstructural destruction in the proximal femur of elderly men are likely concentrated at the superior portion of femoral head, close to the contact site with the medial part of the acetabulum (the socket of the hip joint), the specific “load zone” on the surface of the femoral head that experiences the highest compressive forces and shear stresses during daily activities, affecting the fixation effect and strength of internal fixation for femoral neck fractures (**Figure** **1**A; Figure S1B,C, Supporting Information).

Serum samples were collected from 352 individuals to investigate the association between bone mineral density (BMD) and serum Tes, including 105 young men with normal BMD (Y‐NC), 86 elderly men with normal BMD (E‐NC), 64 elderly men with osteopenia (E‐ON), and 97 elderly men with OP (E‐OP) (Table S1, Supporting Information). Serum levels of Tes, the bone resorption marker type I collagen C‐telopeptide‐related fraction (β‐CTx), and the bone formation marker procollagen type 1 amino‐terminal propeptide (PINP) were measured using Elisa. BMD at the one‐third distal radius was assessed using Dual‐energy X‐ray absorptiometry (DXA). The results showed that β‐CTx levels increased with aging. Compared with elderly men with normal BMD, β‐CTx were significantly elevated in both the E‐ON and E‐OP groups, while no significant difference was observed between the E‐ON and E‐OP. In contrast, PINP levels remained relatively stable (Figure 1A; Figure S1D, Supporting Information). Compared with elderly men with normal BMD, serum Tes declined significantly in the E‐ON and E‐OP groups. There was no significant difference between serum Tes levels in E‐ON and E‐OP. The serum Tes level showed no significant difference between young men and elderly healthy controls (Figure 1B). Linear regression analysis revealed a positive correlation between BMD of the one‐third distal radius and serum Tes levels in elderly men (*n* = 247, Spearman's correlation, *r* = 0.3, *p* < 0.001), whereas no significant correlation was found in young men (*n *= 105, Spearman's correlation, *r* = 0.1, *p* = 0.48), indicating that the association between serum Tes level and bone loss is more significant in elderly men (Figure 1C; Figure S1E, Supporting Information). Receiver operating characteristic (ROC) curve analysis demonstrated an area under the curve (AUC) of 0.65 (95% CI 0.57–0.74, *n* = 86 for healthy controls, *n* = 161 for bone loss) (Figure 1D), suggesting that serum Tes levels may have potential for predicting the risk of bone loss in elderly men. In addition, we analyzed the correlation between serum Tes levels and heel BMD measured by quantitative ultrasound (QUS) in 3,097 individuals from the UK biobank database. Although QUS is less accurate than DXA for BMD assessment, a positive correlation was observed between QUS‐BMD and serum Tes levels (Figure S1F, Supporting Information), consistent with the findings in Figure 1C.

These results highlight the significance of elucidating the mechanism of Tes‐regulated bone remodeling for the early diagnosis and timely treatment for male bone loss.

### 2.2 Tes Stimulates Osteoblast Differentiation and Inhibits Osteoclast Formation, Mainly through Indirect Effects of Osteoblast‐Derived Extracellular Secretions

**Figure 2:**
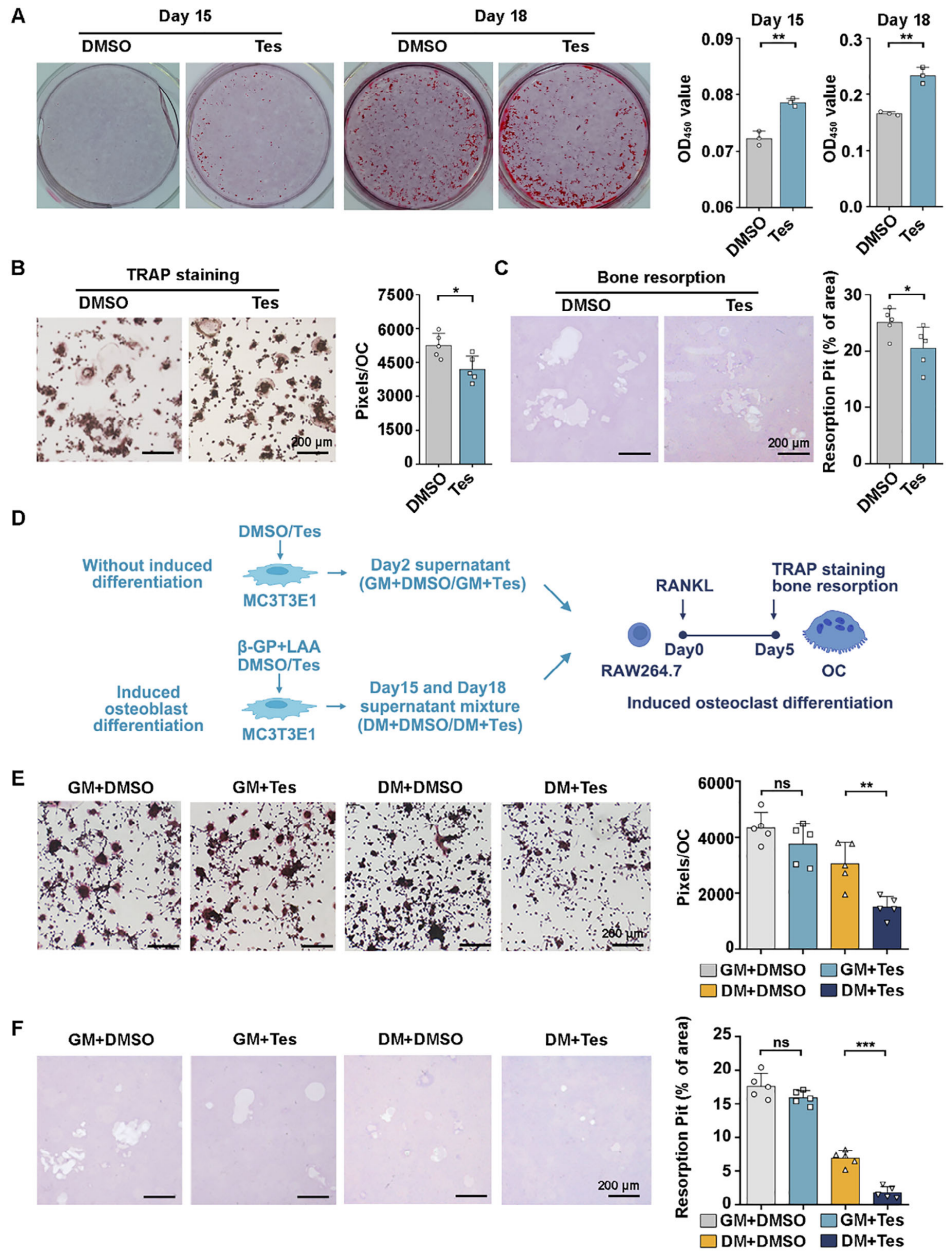
Tes stimulates osteoblast differentiation and inhibits osteoclast formation, mainly through indirect effects of osteoblast‐derived extracellular secretions. A) Representative images and quantification of Alizarin Red staining of MC3T3E1 (osteoblast precursor) cells after 15 and 18 days of osteogenic differentiation in the presence of Tes. B,C) Representative images and quantification of TRAP staining (B) and bone resorption (C) of osteoclasts treated with Tes compared with controls (DMSO). (D) Schematic illustration of the experimental design for Figure 2E,F. E,F) Representative images and quantification of TRAP staining (E) and bone resorption (F) of osteoclasts treated with MC3T3E1‐derived GM or DM supernatant. All results are representative of data from at least three independent experiments. Student's *t*‐test was used for two groups of comparisons, one‐way ANOVA with Tukey's multiple comparisons test was used for multiple comparisons. All tests were two‐sided; *p < 0.05; **p < 0.01; ***p < 0.001; ns = no significance. Tes = testosterone; β‐GP = β‐glycerophosphate; LAA = L‐ascorbic acid; OC = osteoclast; GM = general medium derived from MC3T3E1 culture supernatant; DM = differentiation medium derived from MC3T3E1 culture supernatant. Scale bar, 200 µm.

To investigate the effects of Tes on osteoblast and osteoclast differentiation, an adult male physiological serum concentration of Tes (10^−8^ m) was added to MC3T3E1 (osteoblast precursor, pre‐OB) or RAW264.7 (osteoclast precursor, a monocyte) cells during their differentiation process.^[^ ^25^, ^26^, ^27^ ^]^ The OD_405_ value of Alizarin Red‐positive osteoblasts in cells cultured with Tes was significantly higher than that in controls cultured with DMSO on both Day 15 (metaphase stage) and Day 18 (mature stage) (**Figure** **2**A). These results indicate that Tes continuously promotes osteoblast differentiation with increased production of collagen.

After 5 days of RANKL induction, the formation of mature osteoclasts (>3 nuclei) was confirmed by TRAP staining. The number and rearrangement area of TRAP‐positive (TRAP^+^) multinucleated osteoclasts cultured in the presence of Tes were lower than those of cells cultured with DMSO (Figure 2B; Figure S2A, Supporting Information). Additionally, the area of the pits resorbed on Osteo Assay Surface Plates by TRAP^+^ osteoclasts cultured with Tes was lower than that for cells cultured with DMSO (Figure 2C). Tes exerted moderate inhibitory effects on the phenotypes and bone‐resorptive function of RANKL‐induced osteoclasts.

The interaction between osteoblasts and osteoclasts plays a significant regulatory role in bone remodeling. Osteoblasts can regulate the formation of osteoclasts by secreting various cytokines.^[^ ^28^ ^]^ The direct inhibitory effect of Tes on osteoclasts was relatively mild (Figure 2B,C). We speculate whether Tes regulates osteoclast formation mainly through osteoblasts. The Tes‐free cell supernatant of Tes‐stimulated MC3T3E1 was collected, defined as GM. The Tes‐free cell supernatant of Tes‐stimulated osteoblasts (differentiated from MC3T3E1 cells induced by β‐GP and LAA) at Days 15 and 18 of the differentiation process were separately collected and mixed, defined as DM. To study the effect of Tes through osteoblasts on osteoclasts, GM and DM were used as a conditional medium to cultivate osteoclasts (differentiated from RAW264.7 cells induced by RANKL) (Figure 2D). Under the condition that GM was used as the conditional medium, Tes‐stimulated MC3T3E1 (per‐OB) had no significant impact on the formation of osteoclasts. Under the condition that DM was used as the conditional medium, Tes‐stimulated osteoblasts significantly suppressed the formation of osteoclasts, as the numbers of TRAP^+^ osteoclasts and the rearrangement area were lower (Figure 2E; Figure S2B, Supporting Information). The area of the pits resorbed on Osteo Assay Surface Plates was smaller (Figure 2F). Taken together, our data support a dual role of Tes in protecting bone quality. On one hand, Tes promotes osteoblastic differentiation, and on the other hand, it primarily exerts an inhibitory effect on osteoclasts formation through the indirect effects of osteoblast‐derived extracellular secretions.

### 2.3 Male Mice Lacking AR in Osteoblasts Show Bone Loss and Microstructure Destruction

**Figure 3:**
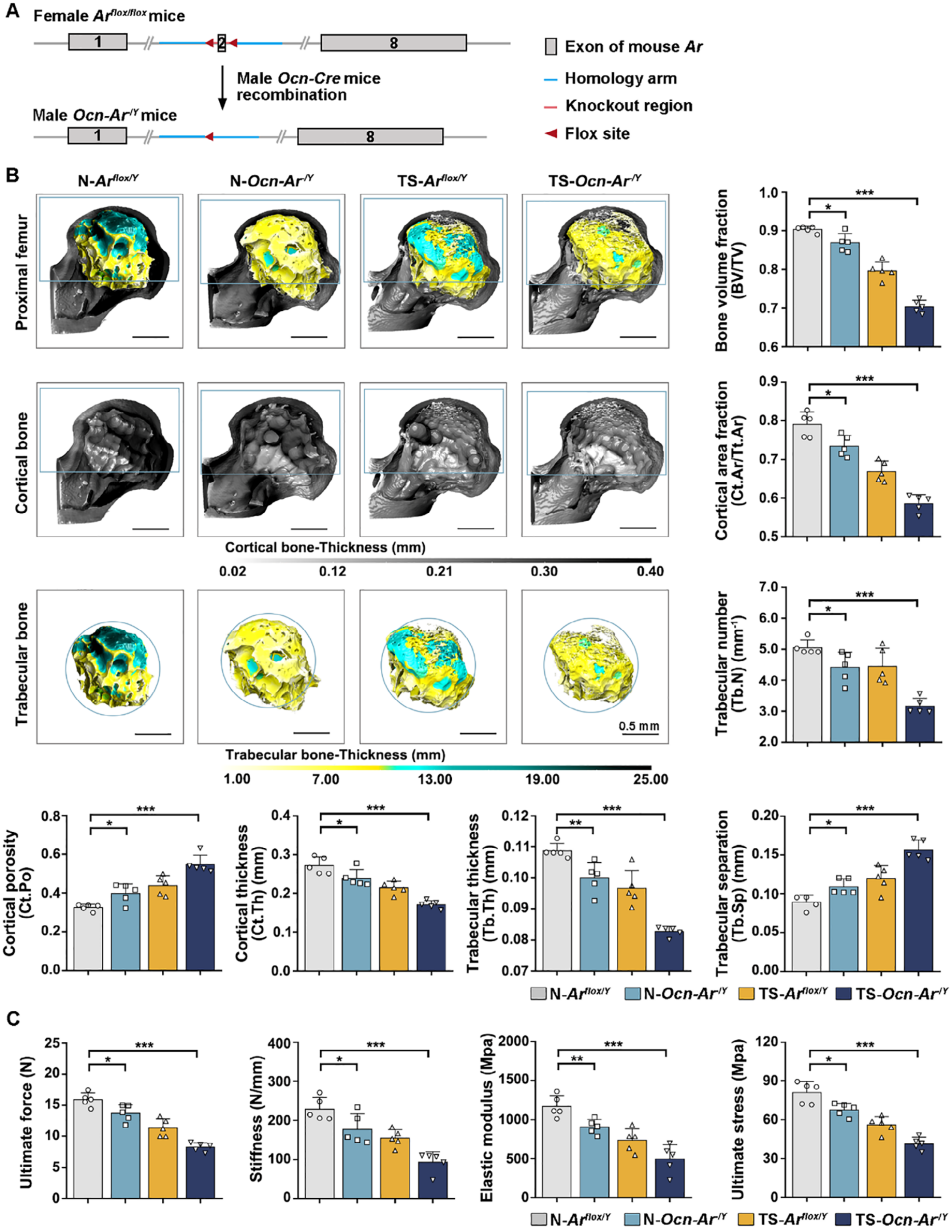
Androgen receptor (Ar)‐deficiency in osteoblasts induces bone loss and accelerates bone destruction in mice. A) Schematic showing the strategy to generate male osteoblast‐specific Ar‐knockout mice (*Ocn‐Ar^−/Y^*). B) 3D reconstruction and bone analysis of micro‐CT images of the proximal femur, cortical bone, and trabecular bone from femurs of *Ocn‐Ar^−/Y^* mice (male, 12‐month‐old) with or without tail suspension; *n *= 5 per group. C) Three‐point bending test, performed to assess the bone strength of the proximal femur of *Ocn‐Ar^−/Y^* mice (male, 12‐month‐old) with or without tail suspension; *n* = 5 per group. One‐way ANOVA with Tukey's multiple comparisons test was used for multiple comparisons. All tests were two‐sided; *p < 0.05; **p < 0.01; ***p < 0.001. *N* = without tail suspension; TS = with tail suspension.

Based on our in vitro findings regarding the indirect effect of Tes‐stimulated osteoblastic inhibition on osteoclasts, *Ocn‐Cre*‐driver (targeting osteoblasts) *Ar*‐knockout (*Ocn‐Ar^−/Y^*) male mice were generated to simulate a low Tes‐AR effect on osteoblast in vivo (**Figure** **3**A). PCR verified the presence of the floxed allele and the Cre transgene in the mouse genotype. Western blot verified *Ar* knockout specifically in osteoblasts (Figure S3A,B, Supporting Information). The body weight increased with age and showed no significant difference between *Ocn‐Ar^−/Y^* male mice and *Ar^flox/Y^* controls (Figure S3C, Supporting Information). However, the BV/TV significantly declined in 12‐month‐old *Ocn‐Ar^−/Y^* male mice compared with *Ar^flox/Y^* controls (declined 4.4%), and the difference was greater at 16 months of age (declined 13.5%). No significant difference was shown between younger (aged 2 and 6 months old) *Ocn‐Ar^−/Y^* male mice and *Ar^flox/Y^* controls (Figure S3D, Supporting Information). These results demonstrate Tes plays a more important role in older mice, which is consistent with the conclusion that Tes is positively related to BMD in elderly men (Figure 1C).

A microgravity OP model was constructed with mice tail suspended for 4 weeks. To clarify the role of Tes through osteoblast‐AR in bone remodeling and bone loss, we not only performed bone analysis on the femurs of *Ocn‐Ar^−/Y^* male mice but also analyzed the femurs of male mice from a new constructed OP model by subjecting the *Ocn‐Ar^−/Y^* male mice to tail suspension for 4 weeks, which resulted in significant bone loss and microstructure destruction. The mice were divided into four groups: *Ar^flox/Y^* mice (N‐*Ar^flox/Y^*), *Ocn‐Ar^−/Y^* mice (N‐*Ocn‐Ar^−/Y^*), *Ar^flox/Y^* mice with tail suspension (TS‐ *Ar^flox/Y^*), and *Ocn‐Ar^−/Y^* mice with tail suspension (TS‐*Ocn‐Ar^−/Y^*). All mice were 12‐month‐old male mice (*n* = 5 per group) and had equivalent baselines of body length and weight (Figure S3E, Supporting Information). The same standard for VOI determination (i.e., the medial part of the trochanter and the area above the lesser trochanter of the femur) as for the hip of elderly men was used in micro‐CT images from the proximal femur of the mice. Dragonfly bone analysis showed a significant decrease in BV/TV, Ct.Ar/Tt.Ar, Ct.Th, Tb.N and Tb.Th, while showing an increase in Ct.Po and Tb.Sp in *Ocn‐Ar^−/Y^* mice. This revealed that *Ar*‐knockout in osteoblasts induced bone loss, decreased the number and thickness of trabecular bone, and increased cortical porosity and trabecular separation at the top position of the proximal femur, which was consistent with the results of Notini^[^ ^29^ ^]^ And Chiang.^[^ ^30^ ^]^ Furthermore, these differences were more significant in TS‐*Ocn‐Ar^−/Y^* mice (Figure 3B), suggesting that osteoblast‐specific Ar knockout combined with tail‐suspension may be an optimized animal model for studying OP in elderly men. The three‐point bending test was performed to evaluate the mechanical characteristics of the proximal femur based on structural changes. The ultimate force, stiffness, elastic modulus, and ultimate stress of TS‐*Ocn‐Ar^−/Y^* mice were significantly reduced compared with N‐*Ar^flox/Y^* controls, and their differences were greater than that between N‐*Ocn‐Ar^−/Y^* mice with N‐*Ar^flox/Y^* controls (Figure 3C).

### 2.4 Extracellular Secreted Proteins TNC is Significantly Upregulated during Tes‐Stimulated Osteoblast Differentiation

**Figure 4:**
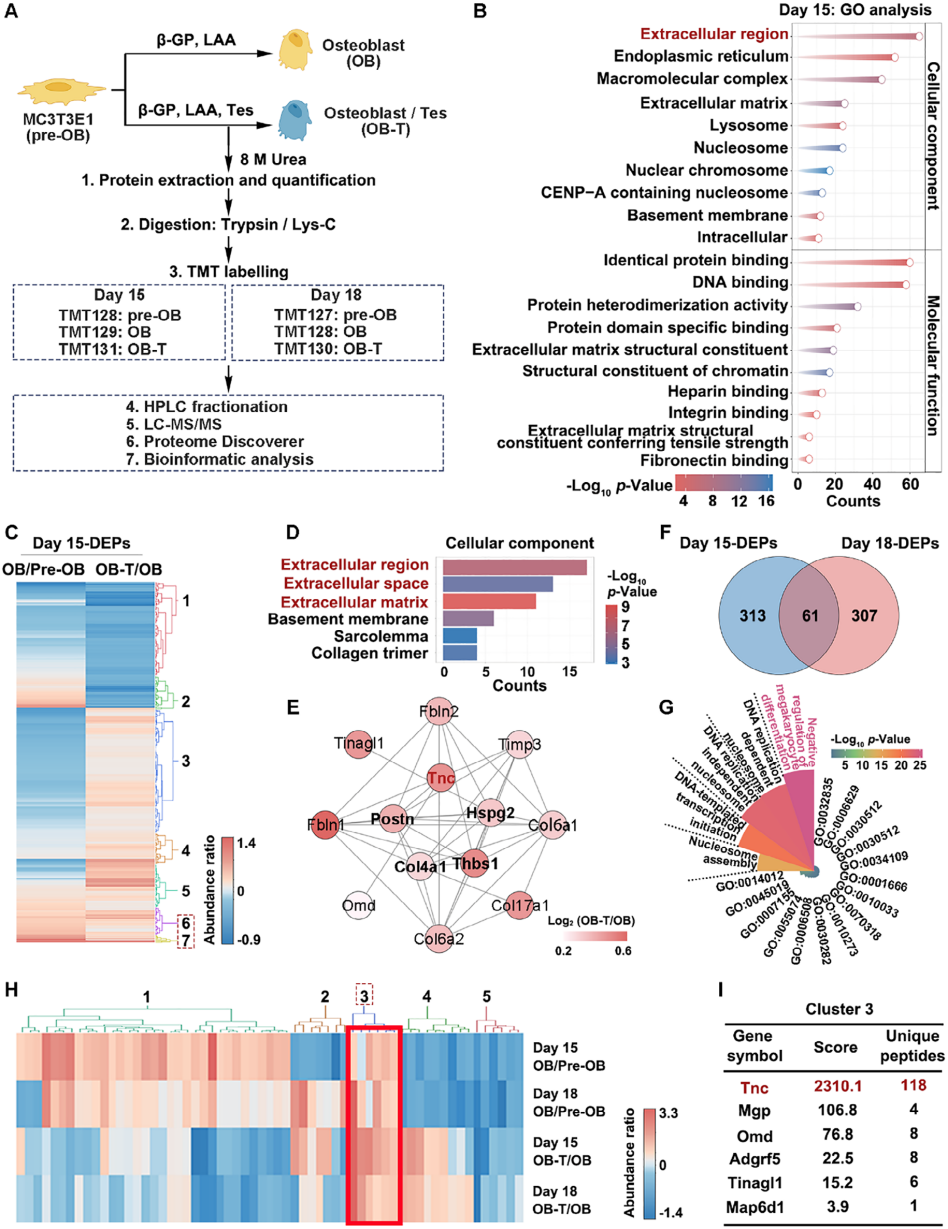
Extracellular secreted protein TNC is significantly upregulated during Tes‐stimulated osteoblast differentiation. A) Flowchart of proteomic experiments. B,C) GO analysis (B) and hierarchical clustering heatmaps (C) of DEPs at day 15 in the process of osteoblast differentiation and Tes‐stimulated osteoblast differentiation. D) Cellular component analysis of Clusters 6 and 7 from panel C. E) STRING analysis of the DEPs from the extracellular region, extracellular space, and extracellular matrix. The center five proteins are hub proteins in the network using the BottleNeck algorithm of the Hubba plug‐in in Cytoscape software. F) Venn diagram showing overlapping DEPs for Tes‐mediated osteogenesis on days 15 and 18. G) Kyoto Encyclopedia of Genes and Genomes enrichment analysis of the 61 overlapping DEPs. H) Hierarchical clustering heatmaps of the 61 overlapping DEPs. I) Mass spectroscopy information for Cluster 3 proteins from panel (H). DEPs = differentially expressed proteins.

As the above findings showed that an indirect inhibitory effect on osteoclast formation through osteoblast‐derived extracellular secretions, tandem mass tag (TMT)‐based quantitative mass spectrometry (MS) was conducted to compare protein changes in pre‐OB, OB, and Tes‐stimulated OB (OB‐T), screening key extracellular proteins secreted from osteoblasts. Two proteomics experiments were performed separately using the OB and OB‐T cells collected on Day 15 and Day 18 of the osteoblast differentiation process (**Figure** **4**A). A total of 8166 proteins were identified in the Day 15 experiment. Among these proteins, 374 differentially expressed proteins (DEPs) were filtered (Figure S4A, Table S2, Supporting Information). A total of 9246 proteins were identified in the Day 18 experiment, and 368 DEPs were filtered (Figure S4A, Table S3, Supporting Information). Gene Ontology analysis was conducted on the DEPs of Day 15 and Day 18. The DEPs were predominantly enriched in the extracellular region, and responsible for identical protein binding and cell adhesion (Figure 4B; Figure S4B, Supporting Information). Hierarchical clustering heatmaps were also constructed and information on proteins in each cluster is shown in Tables S2–S3 (Supporting Information). Protein expression trends in clusters 6 and 7 of Day 15 and cluster 4 of Day 18 showed a sequential increase from pre‐OB to OB, and then to OB‐T, which implies these proteins play important roles in OB differentiation mediated by Tes (Figure 4C; Figure S4C, Supporting Information). These proteins in Day 15 were predominantly enriched in extracellular regions (Figure 4D) and had tight interactions, with five upregulated extracellular proteins identified as central proteins in the interaction network using the cytoHubba plugin of Cytoscape (BottleNeck algorithm, Figure 4E). Among the five central proteins, TNC was also identified in the protein interaction network of DEPs in cluster 4 of Day 18 (Figure S4C, Supporting Information). 61 DEPs overlapped between Tes‐stimulated osteogenesis of Day 15 and 18 (Figure 4F), which were mainly enriched in the negative regulation of megakaryocyte differentiation (Figure 4G, Table S4, Supporting Information). Further hierarchical clustering heatmaps indicated that the DEPs in cluster 3 of overlapped 61 DEPs were consistently upregulated (Figure 4H), with TNC characterized by a high score and a large number of unique peptides (Figure 4I). These results suggest that TNC is likely a key regulatory factor secreted by Tes‐stimulated osteoblasts.

### 2.5 Tes Stimulates Upregulation Of TNC through AR of Osteoblasts

**Figure 5:**
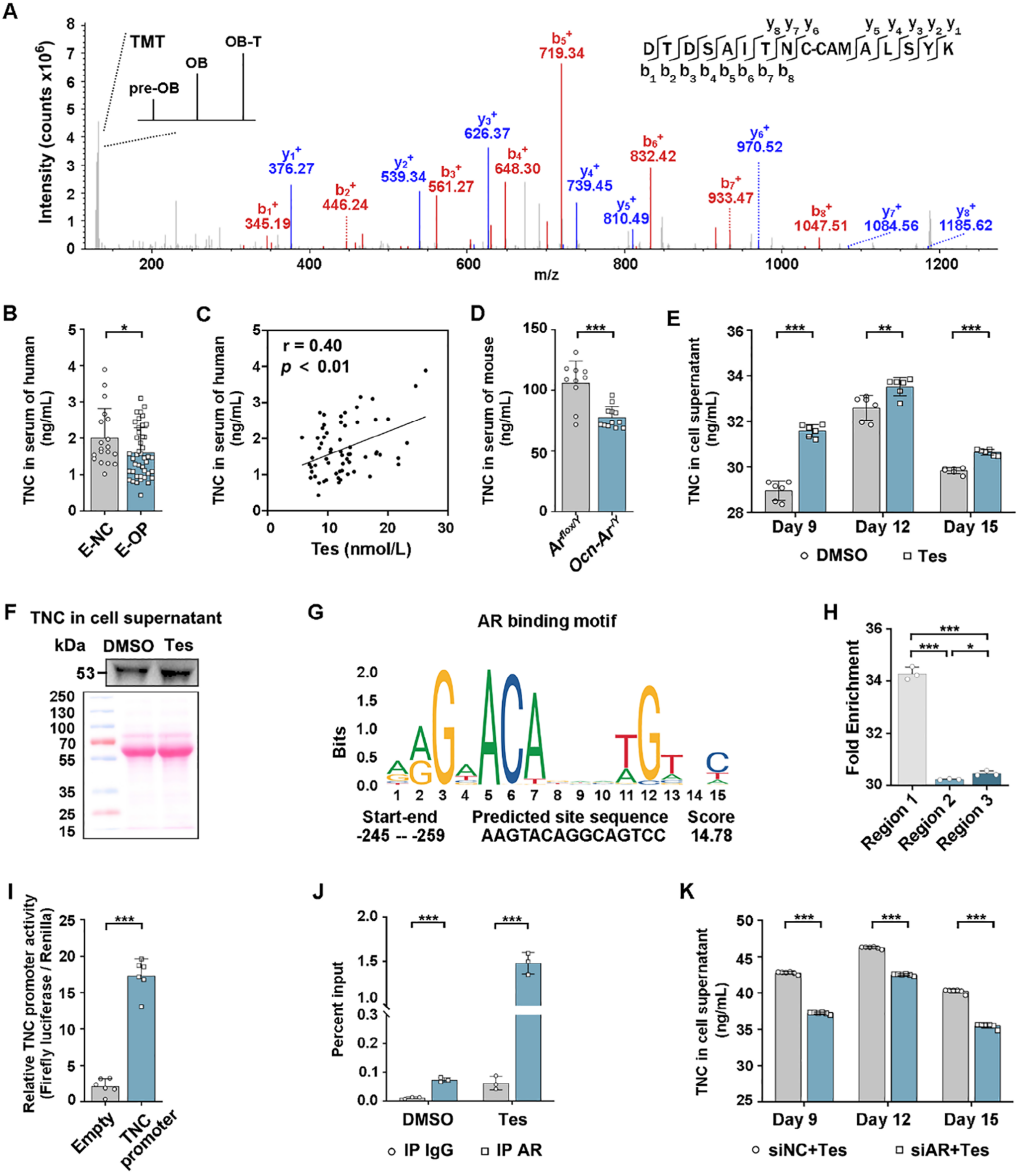
Tes stimulates upregulation of TNC through AR of osteoblast. A) Representative mass spectrum data of TNC. The intensity of TMT precursor ions was used for protein quantification. B) TNC levels in the serum of elderly men with normal BMD (*n* = 19) and elderly men with OP (*n* = 43) by ELISA. C) Correlation analysis between TNC and Tes in the serum in elderly men (*n* = 62). D) TNC levels in the serum of *Ar^flox/Y^* (*n* = 10) and *Ocn‐Ar^−/Y^* (*n* = 12) male mice aged 12 months old by ELISA. E) TNC levels in the cell supernatant of MC3T3E1 on days 9, 12, and 15 in the process of osteogenic differentiation, determined by ELISA to explore the effect of Tes on TNC. F) TNC levels in the cell supernatant of MC3T3E1 on day 15 in the process of osteogenic differentiation detected by Western blot with an antibody targeting the C‐terminus of TNC. The Ponceau S staining was used to verify the sample loading was equal. G) Predicted AR binding sequence in TNC (from the JASPAR website). H) ChIP‐qPCR detection of the binding of AR of different regions of TNC promoter in osteoblast. I) Luciferase reporter assay was performed to determine the transcriptional activation of TNC regulated by AR. J) ChIP‐qPCR detection of the binding of AR in Tes‐stimulated osteoblasts and vehicle‐treated control. K) TNC levels in the cell supernatant of MC3T3E1 in the process of osteogenic differentiation were determined by ELISA. Student's *t*‐test was used for two groups of comparisons, all tests were two‐sided; *p < 0.05; **p < 0.01; ***p < 0.001. E‐NC = elderly men with normal BMD; E‐OP = elderly men with osteoporosis; Tes = testosterone.

The upregulation in TNC expression in OB and OB‐T was verified from the peptide abundances, which were indicated by the intensities of reporter ions (**Figure** **5**A). The serum TNC in elderly men with OP was significantly lower than that in elderly men with normal BMD (Figure 5B) and positively correlated with serum Tes (Figure 5C). The serum TNC in *Ocn‐Ar^−/Y^* male mice was lower compared with *Ar^flox/Y^* controls (Figure 5D), indicating the Tes stimulates upregulation of extracellular TNC through AR of osteoblasts. To verify whether Tes plays a role in the upregulation of extracellular TNC in osteoblasts, supernatants from Days 9, 12, and 15 in the process of osteogenic differentiation were collected. The ALP level of osteoblasts gradually increased beginning on Day 9, and showing that osteoblast differentiation with Tes was higher than that in the control group (Figure S5A, Supporting Information). ELISA identified that TNC in the cell supernatant increased significantly after Tes treatment of osteoblasts, reaching its peak on Day 12; the secretion level decreased on Day 15, but it was still higher in the Tes‐stimulated group than in the control group (Figure 5E). Western blot with an antibody targeting the C‐terminus of TNC showed that expression of TNC in the supernatant of Tes‐stimulated osteoblasts was increased, which was mainly manifested by a band of 53 kDa (Figure 5F; Figure S5B, Supporting Information). To verify that the primary source of TNC is osteoblasts in bone marrow, we performed Immunofluorescence (IF) co‐staining for TNC and OCN in mice femoral head. Co‐staining analysis revealed a significant co‐localization of TNC with OCN^+^ osteoblasts and overlapped with bone trabeculae area. TNC was similarly highly expressed and localized at the bone marrow cavity, suggesting that TNC may act as a secreted protein that is produced by osteoblasts and secreted into the bone marrow (Figure S5C, Supporting Information). ScRNA‐seq data from the NCBI Gene expression omnibus database (GSE253355)^[^ ^31^ ^]^ analysis confirmed single “OCN^+^ Osteoblast” cell had the highest level of TNC expression among 35 clusters in the bone marrow (Osteo‐MSC, Fibro‐MSC, etc.), supporting that “OCN^+^ Osteoblast” is one of the primary source of TNC in bone marrow (Figure S5D,E, Supporting Information).

We explored the mechanism of Tes regulation of TNC. QPCR showed that TNC mRNA levels in Tes‐stimulated osteoblasts were higher than the control group (DMSO), indicating Tes regulated the expression of TNC at the transcriptional level (Figure S5F, Supporting Information). The Jaspar website predicted that AR binding to the TNC promoter utilizes an AAGTACAGGCAGTCC motif with a score of 14.7 (Figure 5G). Using ChIP‐qPCR and dual‐luciferase assay, we found AR bound to region 1 of the TNC promoter (Figure 5H, Text S1, Supporting Information) and increased the TNC promoter activity (Figure 5I). In order to verify the recruitment of AR to region 1 of the TNC promoter was regulated by Tes, we compared the enrichment of region 1 in Tes‐stimulated osteoblasts with vehicle‐treated control. The result showed that AR in Tes‐stimulated osteoblasts bound more DNA fragments derived from region 1 of the TNC promoter (Figure 5J). By silencing of AR in osteoblasts using siRNA (Figure S5G, Supporting Information), the expression of extracellular TNC on Days 9, 12, and 15 of osteogenic differentiation significantly decreased in the cell supernatant (Figure 5K). These results suggest that the Tes‐stimulated upregulation of TNC is through binding with its receptor AR.

### 2.6 The Fibronectin C‐Terminus of TNC Inhibits the Differentiation of Osteoclasts by Binding to Integrin αV and has Therapeutic Effect on Osteoporotic Male Mice

**Figure 6:**
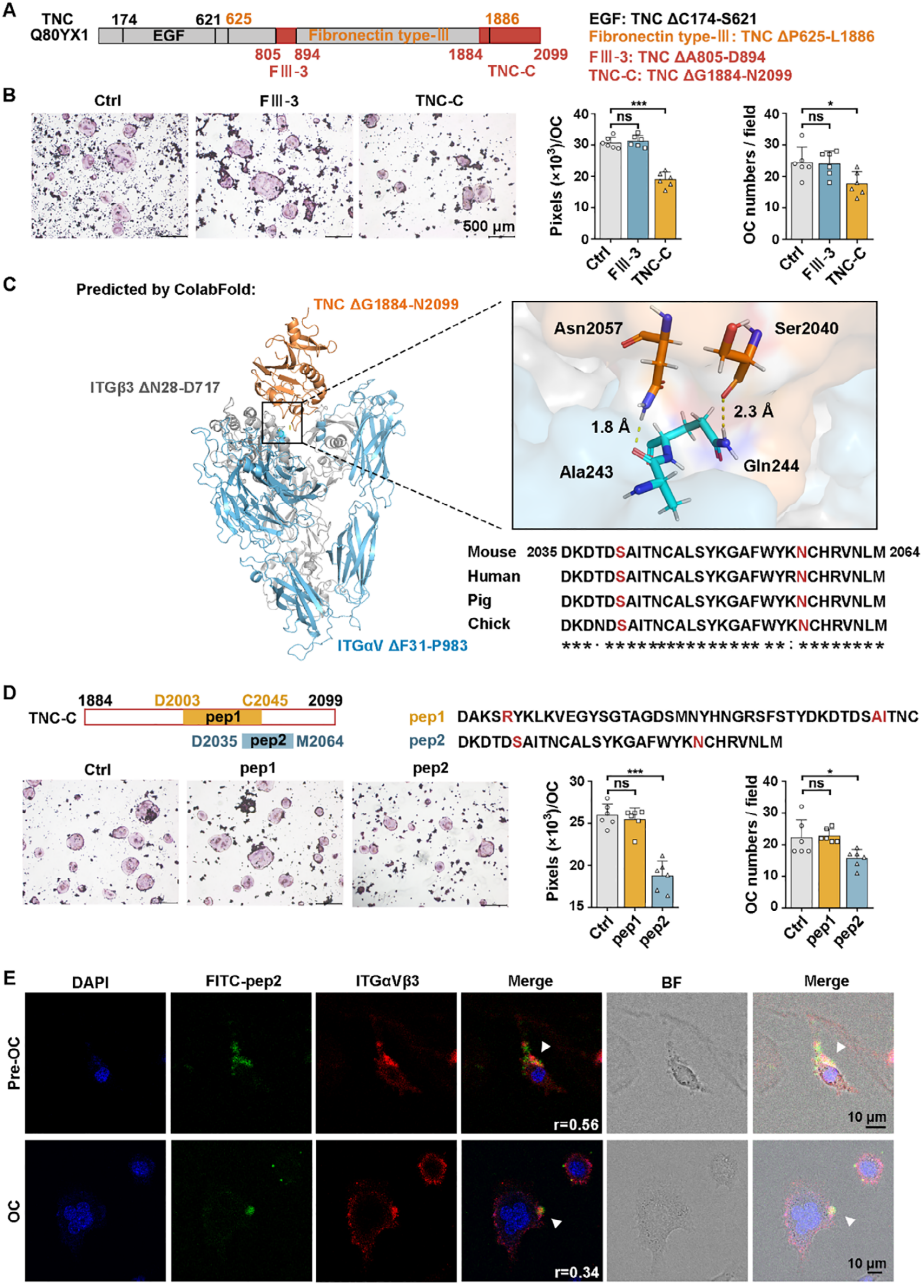
The fibronectin C‐terminus (residues Gly1884‐Asn2099) of TNC (TNC‐C) binds to integrin αV and significantly inhibits the differentiation of osteoclasts. A) Domain structures for TNC. B) TRAP staining was used to detect the inhibition effect of FIII‐3 and TNC‐C on the osteoclast formation. C) Overview of ColabFold docking results for TNC‐C (Gly1884‐Asn2099, orange) with integrins αV (Phe31‐Pro983, blue) and β3 (Asn28‐Asp717, grey) and conservation analysis of the predicted binding sites. D) TRAP staining was used to detect the inhibition effect of peptides from two predicted docking models on osteoclast formation. E) Detection of the localization of FITC‐pep2 and ITGαVβ3 by IF. Student's *t*‐test was used for two groups of comparisons, one‐way ANOVA with Tukey's multiple comparisons test was used for multiple comparisons. All tests were two‐sided; *p < 0.05; ***p < 0.001; ns = no significance. OC = osteoclast; BF = bright field.

TNC has previously been reported to bind to ITGαV‐ITGβ3, which is a transmembrane protein with elevated expression in mature osteoclast differentiation and is believed to bind to non‐collagenous extracellular matrix proteins with RGD motif, such as OPN, to promote the osteoclast differentiation by enhancing adhesion and migration of osteoclast precursor cells on bone absorption site.^[^ ^32^, ^33^ ^]^ The RGD motif is replaced by RVD in mouse TNC.^[^ ^34^ ^]^ There are two RVD motifs in the mouse TNC amino acid sequence, located in the fibronectin type‐III 3 (FIII‐3, TNC ΔA805‐D894) domain and the fibrinogen *C*‐terminal (TNC‐C, TNC ΔG1884‐N2099) domain respectively (**Figure** **6**A). To determine the effects of these two domains on osteoclast formation, we purified recombinant FIII‐3 fragment and TNC‐C fragment from *Escherichia coli* and used them to treat RAW264.7 during the process of osteoclast differentiation. TRAP staining showed that the TNC‐C fragment significantly inhibited the fusion area and number of osteoclasts compared with the FIII‐3 fragment (Figure 6B). In addition, we used an antibody targeting the *C*‐terminus of TNC to block the TNC‐C in the supernatant from Tes‐stimulated osteoblast. The ability of osteoclast differentiation was restored, though it remained lower than that of the group without TES‐stimulated osteoblast supernatant, suggesting that other factors in the osteoblast supernatant also play a role in inhibiting osteoclast differentiation, which may require further exploration in the future (Figure S6A, Supporting Information). Immunoprecipitation assays showed a decrease in the binding of OPN to integrin αV after stimulation with the TNC‐C at various stages of osteoclast differentiation, including RAW264.7 cells, Pre‐OCs (40‐h RANKL‐induced RAW264.7 cells), and OCs, which support the *C*‐terminus of TNC competes with OPN for binding to integrin αV on osteoclasts, thus blocking the adhesion of pro‐osteoclastic proteins (Figure S6B, Supporting Information). To identify the mechanism by which the TNC‐C inhibits osteoclast fusion and key amino acids, ColabFold was used to simulate the docking of the TNC‐C domain and the extracellular region of ITGαV‐ITGβ3. Two docking models were obtained. One model showed the residue Ser2040 of TNC bound to Gln244 of ITGαV and Asn2057 of TNC bound to Ala243 of ITGαV. The two binding sites, residue Ser2040 and Asn2057 of TNC were conserved (Figure 6C). The other model showed the residue Arg2007 of TNC bound to Asp180 of ITGαV, Ala2041, and Ile2042 of TNC bound to Ala245 of ITGαV (Figure S6C, Supporting Information). Based on the results of docking, two peptides including the binding sites were synthesized. Pep1 contains 43 amino acids, located from D2003 to C2045 of TNC protein. Pep2 contains 30 amino acids, located from D2035 to M2064 of TNC protein. TRAP staining demonstrated pep2 is more effective than pep1 as an inhibitor of osteoclast differentiation (Figure 6D). IF staining results showed the colocalization of FITC‐pep2 peptide and ITGαVβ3 (Pearson's correlation coefficient was 0.56 in RAW264.7 and 0.34 in OC), indicating FITC‐pep2 peptide could bound to the ITGαVβ3 (Figure 6E).

**Figure 7:**
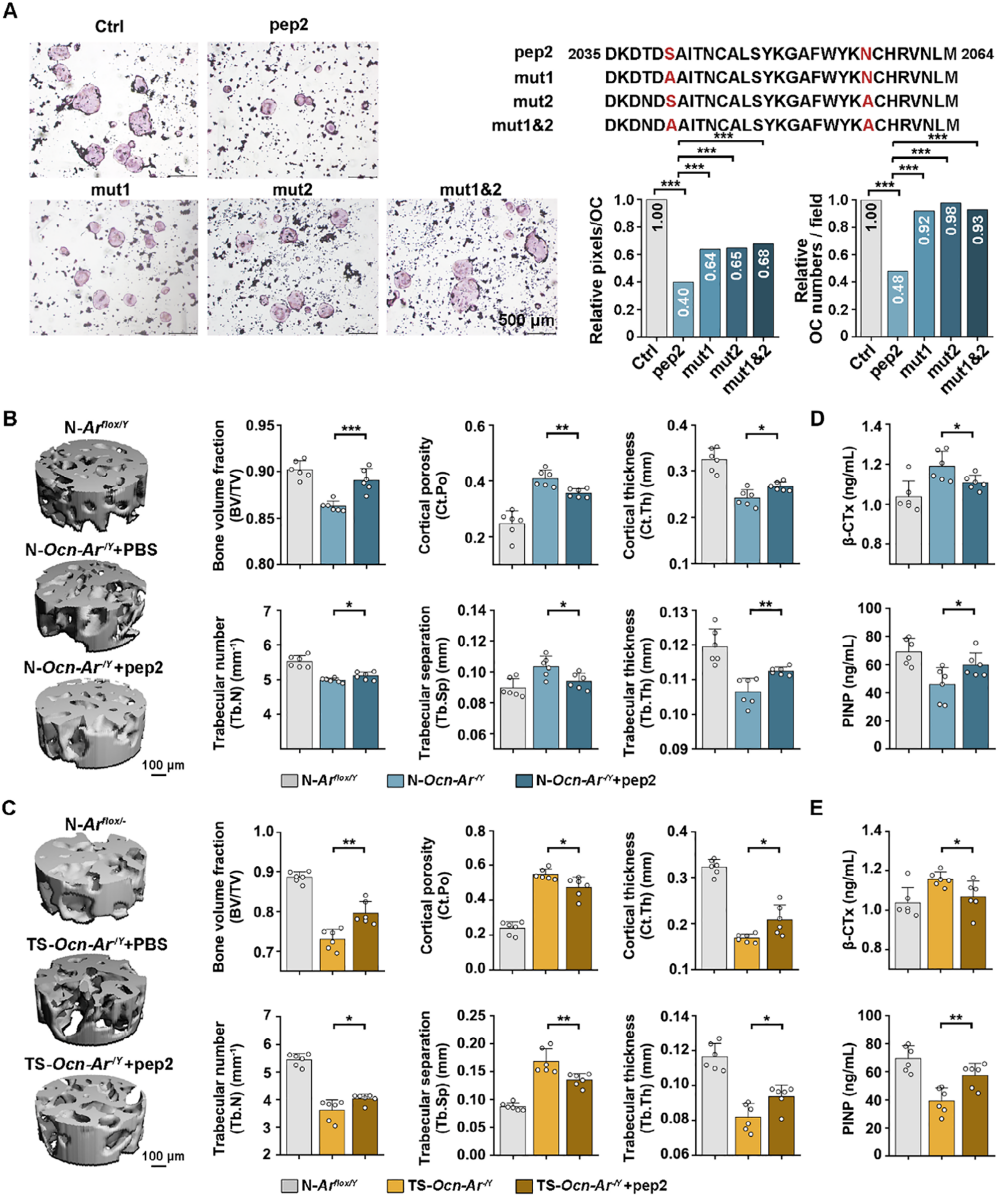
The therapeutic effect of TNC peptides on male elderly osteoporotic mice. A) Evaluation of the importance of the binding sites by detecting the inhibitory effects of mutant peptides on osteoclast formation. B,C) 3D reconstruction and bone analysis of micro‐CT images of the femur of *Ocn‐Ar^−/Y^* mice (male, 14‐month‐old) with or without tail suspension to determine the therapeutic effect of TNC peptides. The serum levels of β‐CTx and PINP in osteoblast‐specific Ar‐knockout OP mice model (D) and osteoblast‐specific Ar‐knockout combined with tail suspension OP mice model (E); *n* = 6 per group. Student's *t*‐test was used for two groups of comparisons, one‐way ANOVA with Tukey's multiple comparisons test was used for multiple comparisons. All tests were two‐sided; *p < 0.05; **p < 0.01; ***p < 0.001.

Mutations were introduced at the two binding sites in pep2 predicted by docking. The mutant peptides showed about a 20% reduction in the ability to inhibit osteoclast fusion and about a 50% reduction in the ability to reduce osteoclast formation (**Figure** **7**A). This suggests Ser2040 and Asn2057 of TNC blocks the binding of other proteins with RGD sequences, such as OPN, to ITGαV‐ITGβ3, thereby inhibiting osteoclast differentiation. In order to explore the feasibility of pep2 in treating OP with low Tes‐AR signaling, 12‐month‐old mice were injected subcutaneously with 80 µg kg^−1^ day^−1^ pep2 or PBS, 5 days per week for 6 weeks (Figure S7, Supporting Information). Micro‐CT and bone analysis were performed to evaluate the therapeutic efficacy of pep2. For both the osteoblast‐specific *Ar*‐knockout OP mice model and the osteoblast‐specific *Ar*‐knockout combined with tail suspension OP mice model, the femur of the pep2‐treated mice had greater BV/TV, Ct.Ar/Tt.Ar, Ct.Th, Tb.N and Tb.Th than those of the *Ocn‐Ar^−/Y^* controls (Figure 7B,C; Figure S7B,C, Supporting Information). To assess the therapeutic effect of pep2 on bone turnover, serum markers of bone resorption and bone formation were analyzed. The results showed a significant reduction in β‐CTx serum level after pep2 treatment. Additionally, serum levels of the bone formation marker PINP were significantly increased (Figure 7D,E). These data demonstrating the pep2 therapeutic effect on OP, further investigation of the pharmacokinetics of the TNC peptides would help to further clarify the dose and course of polypeptide therapy.

### 2.7 SEV‐APP Combined with Tes Level is a Potential Biomarker in Serum for Early Risk Assessment of OP in Elderly Men

**Figure 8:**
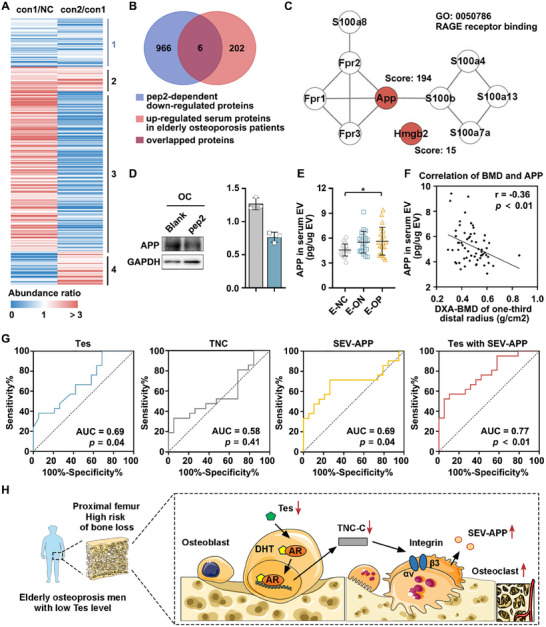
SEV‐APP combined with Tes level is a potential biomarker in serum for early risk assessment of OP in elderly men. A) Hierarchical clustering heatmaps of proteins of cell lysates of osteoclasts and osteoclasts stimulated by 10 or 20 ng mL^−1^ pep2. B) Venn diagram showing overlapping proteins. C) STRING analysis of the proteins in RAGE receptor binding. D) Verification of APP expression in the cell lysates of osteoclasts stimulated by pep2 by Western blot. E) Comparisons of SEV‐APP level in elderly men with normal BMD (E‐NC, *n* = 19), elderly men with osteopenia (E‐ON, *n* = 21), and elderly men with osteoporosis (E‐OP, *n* = 21) by ELISA. F) Correlation analysis between SEV‐APP and DXA‐BMD of one‐third distal radius in elderly men (*n *= 61). G) ROC curve analysis to evaluate the effectiveness of diagnosing OP (*n *= 19 for normal BMD; *n* = 21 for OP). H) Graphical abstract of the study. One‐way ANOVA with Tukey's multiple comparisons test was used for multiple comparisons. All tests were two‐sided; *p < 0.05. OC = osteoclasts; EV = extracellular vesicles; DXA = dual‐energy X‐ray absorptiometry; BMD = bone mineral density; Tes = testosterone.

In addition to the potential treatment of TNC in OP, could TNC also be useful as a biomarker for the auxiliary diagnosis of OP? The ROC curve analysis showed that TNC was not effective in diagnosing OP (Figure S8A, Supporting Information, *p *= 0.05). In order to identified effective biomarkers, the cell lysates of osteoclasts (NC), 10 ng mL^−1^ (con1), and 20 ng mL^−1^ (con2) pep2 peptide‐stimulated osteoclasts were collected to performed the proteomics. A total of 5371 high‐confidence proteins were identified (Table S5, Supporting Information). By defining proteins with a ratio greater than 1 as an upregulated trend, and conversely, proteins with a ratio less than 1 as a downregulated trend, the identified proteins were divided into 4 Clusters based on the change trend (**Figure** **8**A). Cluster 1 comprised 972 proteins that exhibited a pep2 concentration‐dependent downregulation pattern. This consistent dose‐response relationship suggests these proteins are likely regulated by TNC and may participate in osteoclast differentiation and function. Our previous proteomics study found that 208 proteins in serum‐derived extracellular vesicles (SEV) were increased in elderly men with OP.^[^ ^35^ ^]^ The Venn diagram showed there were 6 proteins overlapped (Figure 8B). Among the 6 proteins, APP (protein identification confident score is 194) and Hmgb2 (protein identification confident score is 15) were enriched in the RAGE receptor binding network. Several studies demonstrated that RAGE (abbreviation of the receptor for advanced glycation end products) plays a role in bone homeostasis and can contribute to the onset of several bone‐related diseases, including OP.^[^ ^36^ ^]^ The protein–protein interaction analyzed by STRING showed APP occupied a central position in the network (Figure 8C). The result of the western blot indicated the expression of APP in pep2‐stimulated osteoclasts was significant reduced (Figure 8D). We also used ELISA to detect the APP in serum EV of mice after pep2 treatment. The results showed that regardless of whether combined with tail suspension, the APP in serum EV of osteoblast‐specific AR knockout mice were increased and decreased significantly after treatment with pep2 peptide (Figure S8B,C, Supporting Information). The result of ELISA showed the SEV‐APP level gradually increased in the elderly healthy men, elderly ON men, and elderly OP men (Figure 8E). These results were consistent with the mass spectrometry data. Correlation analysis showed a negative correlation between SEV‐APP and BMD in elderly men (Figure 8F). We further analyzed the potential clinical significance of SEV‐APP. ROC curves were performed to compare in detail the effectiveness of Tes, TNC, APP, in combination of two markers and in combination of all three markers in diagnosing OP. The results demonstrated the AUC of Tes combined with SEV‐APP was 0.77, and the AUC of the combination of all three markers was also 0.77, which was higher than that of others (Figure 8G; Figure S8D–F, Supporting Information). Based on these results, the Tes combined with SEV‐APP thought to have potential clinical value in assisting the early risk assessment and diagnosis of OP. A graphical abstract of this study is shown in Figure 8H, demonstrating that Tes suppresses the overactivation of osteoclast‐mediated bone degeneration in elderly men via osteoblast‐AR‐mediated upregulation of the fibronectin *C*‐terminus of TNC.

## 3 Discussion

Although the beneficial effects of sex hormones on bone have been reported widely, the elucidation of the molecular mechanisms underlying the roles of androgen in bone structure and strength is still incompletely understood.^[^ ^37^ ^]^ Estrogen deficiency is commonly accepted to be a critical factor in the degeneration of the skeleton in both sexes; Vanderschueren et al. reported that lack of bioavailable β‐E2 could potentially play a role in the development of OP in aging men.^[^ ^38^ ^]^ However, the estrogen‐centric view of bone remodeling is increasingly being challenged by a greater understanding of the pathogenesis of bone loss. Although a legitimate argument can be made that the effects of androgen on bone are mediated principally by the aromatization of estrogen,^[^ ^10^ ^]^ accumulating evidence indicates that androgens exert direct effects in the maintenance of body composition and trabecular parameters.^[^ ^39^ ^]^

It remains likely that Tes impacts bone size by influencing periosteal apposition and, potentially indirectly, bone mass by augmenting muscle mass.^[^ ^19^ ^]^ As individuals age, it is typical for Tes levels to decline gradually. However, there should not be an abrupt drop in Tes levels in men that parallels the drastic decrease in estrogen observed during menopause in women.^[^ ^7^ ^]^ There is an immediate decline in Tes levels due to androgen deprivation therapy (ADT) for prostate cancer, leading to bone loss. Typically, BMD decreases by ≈2–8% within the first year after starting ADT. While TRT is thought to help preserve and even increase BMD in hypogonadal men, there is currently no evidence to support the idea that TRT can prevent bone fractures. Presently, TRT is not recommended solely for the purpose of enhancing and maintaining BMD in hypogonadal men. Rather, it should be considered as one of the treatment options for improving both hypogonadal symptoms and BMD simultaneously in symptomatic hypogonadal men with ON.^[^ ^8^ ^]^ Our study identifies that BMD is positively correlated with the serum Tes level in elderly men, and the cut‐off value of the ROC curve for evaluation of bone loss in elderly men was 9.415 nmol L^−1^ (normal range in men > 60‐years‐old 3.02–27.07 nmol L^−1^). That is, a serum Tes level < 9.415 nmol L^−1^ in elderly men is a negative factor for bone health, and there is a risk of bone loss. Close monitoring of bone health and active intervention in terms of lifestyle, exercise, and fall prevention are needed. However, the lack of deep understanding of the mechanisms by which Tes protects bone quality and bone strength still restricts early diagnosis and precise intervention in bone loss in elderly men.

To investigate the role of Tes in OP among elderly men, we employed a mouse model for in vivo studies. OP mouse models are broadly divided into two types: reduced bone formation models, such as the senile osteoporotic model for aging‐related OP, and increased bone resorption models, such as ovariectomized osteoporotic model, the gold standard for postmenopausal OP research, and the disuse model for OP due to limited or impaired physical activity. Additionally, genetic engineering‐based OP models are in the exploratory stage. Current researches show that knockout of genes such as OPG, RUNX2, and TGF‐β1 can induce OP models. Our study focuses on the effects of Tes on the development and progression of OP in elderly men. Existing animal models do not sufficiently simulate the effect of low Tes on OP in elderly men. Based on findings that Tes inhibits osteoclast formation through osteoblasts, we constructed osteoblast‐specific AR knockout mice. Analysis of the femur in 12‐month‐old mice indicates that osteoblast‐specific AR knockout in elderly male mice can induce OP, representing a new modeling approach. Furthermore, considering the decreased mobility in the elderly, we combined the osteoblast‐specific AR knockout with the disuse osteoporotic tail suspension model. This combination results in a more significant reduction in bone mass. The use of old male mice with osteoblast‐specific AR knockout combined with the tail suspension serves as an animal model to investigate the pathogenesis of OP associated with age‐related Tes decline in elderly males and to identify effective therapeutic strategies. However, humans are the only primate species in nature that rely primarily on fully upright walking as their main mode of locomotion. The load‐bearing mechanisms in existing animal models cannot fully replicate the biomechanical forces exerted on the human femoral head during upright walking.

Our data from experiments in mice demonstrate that Tes action via the AR regulates trabecular bone remodeling, which is consistent with research in humans showing that maintenance of serum Tes levels but loss of estrogen production led to significant decreases in cortical area and thickness (with increases in Ct.Po). Khosla et al. estimated that of the total effect of sex steroids on bone resorption in men, estrogen accounted for 70% and Tes for at most 30%.^[^ ^40^ ^]^ However, estrogen levels in men are consistently low (73.42–172.837 pmol L^−1^) and are associated with estrogen‐only when they are <40 pmol L^−1^.^[^ ^41^ ^]^ This parsing of the resorption data to reflect estrogen effects on cortical bone versus Tes effects on trabecular bone (70:30) is remarkably consistent with the fact that the skeleton is ≈80% cortical bone and ≈20% trabecular bone.^[^ ^42^ ^]^ Therefore, we speculate that bone loss in elderly men, especially the destruction of bone microstructure, is closely related to the weakening of the regulatory Tes‐AR signal of osteoblasts, and the effect of enhancement of osteoclast activity on trabecular bone, and its regulatory mechanism needs to be elucidated. In the present study, analysis of bone microarchitecture indicated that bone destruction in the proximal femur of elderly males is mainly manifested as a decrease in the thickness of cortical bone and trabecular bone and an increase in the Ct.Po at the superior portion of the femoral head, accompanied by a reduction in the serum Tes level. In vivo studies in mice lacking AR in osteoblasts identified that Ct.Po and Tb.Sp were increased at the top of the proximal femur after tail suspension.

For clinical significance, the medial cortex at the junction area of the femoral head and femoral neck, in addition to the trabecula in the subchondral bone of the femoral head, are of great account for the biomechanical stability of internal fixation (either the femoral neck system or cannulated cancellous screws) for femoral neck fractures.^[^ ^43^ ^]^ It is true that the femoral neck is much more important as a fracture site, while in view of the poor prognosis and easy failure of surgery, the present study is more focused on the mechanical loading area for internal fixation, and the underlying molecular regulatory path of Tes in bone remodeling of aged men with OP. Hip fractures in the elderly include two major diseases, femoral neck fracture and intertrochanteric fracture, and one of the commonly used hip‐preserving surgeries is hollow nail fixation and the other is proximal femoral nail antirotation. Both procedures require strong bony support in the subchondral bone of the femoral head and the Adam's arch of the femoral neck, otherwise, serious complications such as surgical failure, delayed fracture healing, and re‐fracture are likely to occur. Thus, the VOI was determined as which includes the complete coronal position of the femoral head, and the medial part of the trochanter, and the area above the lesser trochanter of the femur. As we previously reported, the integrity of these structures determines the effectiveness of surgical treatment,^[^ ^44^, ^45^ ^]^ suggesting that identification of the underlying mechanisms in bone loss and microstructural destruction is conducive to improve the prognosis and reduce the failure rate of surgery of those elderly men with low Tes levels.

Research findings indicate that β‐E2 significantly contributes (≈70%) to the inhibition of osteoclast formation through the induction of Fas ligand expression in osteoblasts.^[^ ^46^ ^]^ While Tes inhibits osteoclastogenesis by ≈30%, the specific mechanism through which it does so remains unidentified.^[^ ^15^ ^]^ There is limited evidence to support the hypothesis that Tes directly affects osteoclasts.^[^ ^8^ ^]^ Our in vivo studies suggested that the loss of Tes‐AR signaling in osteoblasts leads to accelerated bone destruction under microgravity‐induced osteoclast overactivation. Before the in vitro studies, we searched the literature and found the Tes concentration in male blood is equivalently to the concentration level 10^−8^ m.^[^ ^25^, ^26^, ^27^ ^]^ The concentrations of 10^−8^ m Tes and 10^−9^ m DHT had similar significant promoting effects on osteoblasts (Figure S9, Supporting Information). In vitro study identified that Tes activates osteoblast‐AR‐mediated upregulation of the fibrinogen *C*‐terminus of TNC during the differentiation of osteoblasts, which inhibits osteoclast differentiation and the area of osteoclast fusion. ITGαV‐ITGβ3 is known to be highly expressed in fully differentiated and mature osteoclasts;^[^ ^47^ ^]^ it engages in osteoclast migration, adhesion, and fusion by binding to the colony‐stimulating factor‐1 receptor, thereby initiating cytoskeletal formation and activating the ERK/c‐Fos signaling pathway.^[^ ^48^ ^]^ Antagonists targeting ITGαV‐ITGβ3 are regarded as effective pharmaceuticals for postmenopausal OP.^[^ ^49^ ^]^ A binding site within the FN III repeat of TNC has been identified for ITGαV‐ITGβ3, which contains a cryptic RGD sequence.^[^ ^32^ ^]^ This sequence is typically obscured by the adjacent second FN III repeat.^[^ ^50^ ^]^ Another binding site for ITGαV‐ITGβ3 has been located within the *C*‐terminal FBG region of TNC. This site is responsible for the diminished adhesion of endothelial cells to TNC substrates.^[^ ^32^, ^51^ ^]^ However, the precise binding site and mechanism of action in ITGαV‐ITGβ3‐expressing cells of the *C*‐terminal FBG of TNC have not yet been explored in detail.^[^ ^51^ ^]^ We noticed that the focal binding of FITC‐pep2 with ITGαV‐ITGβ3 demonstrating a sign of polarization, which is not uniformly consistent. On one hand, there are different kinds of integrins (including but not limited to αVβ3, α2β1 , and αVβ5) are expressed in the process of osteoclast differentiation and maturation, and their expression on the osteoclast cell membrane is dynamically changing. On the other hand, the highly selective binding domain in TNC‐C pep2 can probably change the distributions of ITGαV‐ITGβ3.^[^ ^48^, ^52^, ^53^ ^]^

In these in vitro studies, we used murine‐derived osteoblasts and osteoclasts. The conclusions may be somewhat inadequate to reflect the real situation in the human body. Both human and mouse cell lines have been artificially modified and cannot fully reflect the real situation in the body. While the DNA binding domain of AR is the most highly conserved region between the different members of the steroid hormone nuclear receptor family.^[^ ^54^, ^55^ ^]^ Perhaps we need to develop models that are closer to what the human body really looks like, such as bone organoids. However, at present, the construction of bone organoids still faces challenges, such as the types, sources, identification, and separation methods of bone stem cells, and how to select and optimize the extracellular matrix suitable for the construction of bone organoids still needs further research.^[^ ^56^, ^57^ ^]^

ColabFold was used in our study to simulate the docking of the fibrinogen *C*‐terminus of TNC with the extracellular regions of ITGαV‐ITGβ3. The fibronectin *C*‐terminus thus blocks the binding of other proteins with RGD sequences to ITGαV, thereby inhibiting osteoclast fusion and bone resorption. On the contrary, full‐length TNC has been noted to facilitate cartilage repair and deter the onset of osteoarthritis without worsening synovitis.^[^ ^58^ ^]^ Meanwhile, alternative research identified TNC as an endogenous danger signal responsible for driving TLR4‐dependent fibroblast activation. Moreover, its persistence impedes the resolution of fibrosis.^[^ ^59^ ^]^ Hence, considering the structural, expression, and functional traits of TNC, the alternative splicing variants of TNC assume diverse roles in various physiological and pathological conditions.^[^ ^23^ ^]^

Research findings also demonstrate a negative relationship between serum Tes and TNC among young men diagnosed with major depressive disorder (average age: 36.48 ± 15.73),^[^ ^60^, ^61^ ^]^ which indirectly confirms our findings that Tes and TNC pose multiple correlations. Notably, young men exhibit elevated Tes levels compared to elderly men, with both control and major depressive disorder groups displaying higher levels than those observed in the elderly men included in our study. While major depressive disorder and OP represent distinct conditions, they may share common underlying mechanisms. The mouse tail suspension model serves not only as a model for OP but also as a model for depressive disorder. Combining our findings investigated from human samples and in vivo mouse models, Tes and TNC might exert regulatory influences on both OP and major depressive disorder. However, a concentration‐dependent shift is very likely existing in the regulatory effect of Tes on TNC, resulting from the neuro‐bone tissue specificity and distribution of androgen receptors, which play a distinct role in the mechanisms underlying various diseases.

In terms of our investigation, lack of AR‐Tes signaling would lead to downregulation of the extracellular fibrinogen *C*‐terminus of TNC, indirectly inhibiting osteoclastogenesis and bone resorption. Thereby, the osteoclastic overactivation resulting from physical inactivity is disinhibited. Combined with the decreased osteoblastic bone formation, a net decrease in cortical and trabecular thickness occurs, decreasing bone strength and increasing fracture risk.^[^ ^62^ ^]^ This can explain why some elderly men lose bone mass rapidly when they are bedridden or have limited activities. Although most men will not suffer from extreme hypogonadism, as they age, the Tes levels of some men drop to lower than normal levels.^[^ ^63^ ^]^ When this group of people undertake limited activities, significantly reduce weight‐bearing exercises, or even become bedridden due to advanced age or injury, rapid osteoclast activation and bone resorption cannot be limited by the fibrinogen *C*‐terminus of TNC secreted by osteoblasts, resulting in a reduction in the thickness of the bone cortex and trabecular bone, and a reduction in the density of trabecular bone connections, thereby lowering bone strength. Our data demonstrating the TNC‐C pep2 therapeutic effect on bone loss and destruction of those elderly men with low Tes, while numerous investigations including chemistry, manufacturing and control and pharmacokinetics of the peptides would to be done before it processes to clinical application.

Osteoclast activity results in the breakdown of the bone matrix, leading to the release of specific biochemical markers into the bloodstream.^[^ ^64^ ^]^ These include *C*‐terminal telopeptide (CTX) and *N*‐terminal telopeptide (NTX), products of type I collagen, which are indicative of osteoclastic activity and bone resorption.^[^ ^65^ ^]^ They are commonly used to assess the risk of OP and to monitor the efficacy of OP treatment, but cannot be used alone to diagnose or differentiate OP from normal bone.^[^ ^66^ ^]^ In the present study, we combined the SEV‐APP and serum Tes levels in elderly males to determine senile bone destruction—the results significantly improved the accuracy of identifying the elderly population at high risk of bone destruction. This is a new strategy for advancing the diagnostic window of bone destruction assessment in elderly men, realizing early and accurate intervention, and evaluating treatment prognosis. In addition, we also paid attention to the bone nonunion (a fracture that persists for a minimum of 9 months without signs of healing for 3 months), which means the body's inability to heal a fracture and causes devastating outcomes for patients. The ability to promptly identify patients at high risk for nonunion will allow for the success of early appropriate targeted therapeutic intervention. Research and progress have been made in understanding the pathophysiological processes of bone therapy to deal with markers of increased nonunion susceptibility. Moghaddam et al. found that patients with atrophic non‐union exhibited significantly lower serum levels of CTX in the first week and reduced TRACP 5b at weeks 4 and 8.^[^ ^67^ ^]^ Zimmermann et al. found that while TGF‐β1 levels initially rise after a fracture, they decline earlier in patients with delayed bone healing.^[^ ^68^ ^]^ In our study, we evaluated the ability of Tes combined with SEV‐APP to predict the risk of bone nonunion following femoral neck fractures. According to the cutoff value of Tes (9.985 nmol L^−1^) and SEV‐APP (4.868 pg µg^−1^), elderly (>60 years) men with OP were divided into four groups (Figure S10A, Supporting Information, *n* = 20 per group). The occurrence of bone nonunion in elderly OP men with Tes level below 9.985 nmol L^−1^ and SEV‐APP level above 4.868 pg µg^−1^ (Figure S10B, Supporting Information), indicating Tes combined SEV‐APP might be a potential biomarker for predicting the bone nonunion. Recent studies have focused on how the APP and its metabolites affect bone cell function. APP is a transmembrane protein that can be cleaved to produce amyloid‐beta peptides. It has been found that APP and its derivatives can regulate the differentiation and activity of osteoblasts and osteoclasts. APP can either enhance or inhibit Wnt‐mediated signaling in osteoblasts, depending on the specific context and the presence of other regulatory factors.^[^ ^69^ ^]^ The emerging concept of the “bone–brain axis” highlights the communication between the skeletal system and the nervous system. APP, given its role in neurodegenerative diseases and its potential impact on bone cells, is being investigated as a key player in this crosstalk.^[^ ^70^ ^]^ However, the presented available data could only serve as preliminary findings toward this goal. Further large‐scale clinical studies are required. For example, quantitative analysis of specific APP‐derived peptides in the blood or cerebrospinal fluid may provide insights into the risk of bone loss and the progression of OP.

In this study, neither a single‐center validation using the primary index of DXA‐BMD of one‐third distal radius nor the secondary validation with QUS‐BMD of heel bone collected from UK Biobank could fully satisfy our ultimate goal of studying the DXA‐BMD of the proximal femur. Compared with DXA, QUS has the advantages of portability, low cost, and no radiation,^[^ ^71^ ^]^ which can obtain a larger sample size. However, due to the impact of soft tissue and bone redundancy, and the diagnostic accuracy of QUS in the heel bone is lower than that of DXA and the long‐term follow‐up capability is still unclear.^[^ ^72^ ^]^ Although DXA‐BMD of the one‐third distal radius may provide useful clinically information, its predominantly cortical bone component may not fully reflect metabolic changes in trabecular bone. Compared with the proximal femoral site, one‐third distal radius measurements may be less sensitive for early osteoporosis detection^[^ ^73^ ^]^ and can only meet our needs to a certain extent. In addition, the cortical bone of mice is quite different from that of humans, because humans have extensive intracortical osteonal remodeling that is absent from mice.^[^ ^19^ ^]^ Thus, the study of the regulation of bone microstructure by Tes, particularly in the trabecular bone of elderly men, requires sufficient quantity and higher‐resolution CT images combined with prudent interpretations from mouse models.

## 4 Conclusion

In conclusion, this study reveals that Tes‐AR signaling delays elderly male bone microarchitectural destruction by upregulation of the osteoblastic TNC, particularly the fibrinogen C‐terminus, exerts a suppressive effect on osteoclastogenesis by competitively binding to integrin αV, thereby interfering with pro‐osteoclastic signaling. The Tes‐AR‐TNC axis emerges as a central regulatory pathway in preserving bone microarchitecture under age‐related or mechanical unloading conditions. Moreover, the identification of APP in extracellular vesicles as a biomarker linked to AR‐TNC axis deficiency provides a potential diagnostic biomarker for early detection of bone loss. These findings offer mechanistic insights and highlight novel targets for precision prevention and treatment of bone loss and nonunion.

## 5 Experimental Section

### Reagents

Reagents and kits were purchased from commercial sources. Iodoacetamide (IAA), dithiothreitol (DTT), urea, and cell culture medium were from GE Healthcare (Little Chalfont, Bucks, UK). The BCA protein assay kit, TMT labeling kits, and fetal bovine serum (FBS) were from Thermo Fisher Scientific (Waltham, MA, USA), and the protease inhibitor cocktail was from Roche (Basel, Switzerland). Sequencing‐grade endoproteinase Trypsin/Lys‐C was from Promega (Madison, WI, USA). Ascorbic acid‐2‐phosphate (LAA), β‐glycerol phosphate (β‐GP), β‐E2, Tes, the TRAP, and the Alizarin red staining kits were purchased from Sigma (St. Louis, MO, USA). An Xbridge BEH300 C18 column (4.6 × 250 mm, 5 µm) was obtained from Waters (Milford, MA, USA). A fused silica capillary column (75 µm ID, 150 mm length) was purchased from Upchurch (Oak Harbor, WA, USA), and C18 resin (300 A, 5 µm) was from Varian (Palo Alto, CA, USA).

### Serum Samples and Computed Tomography (CT) Images of Recruited Individuals

Elderly (aged above 60 years) and young (aged below 60 years) men diagnosed with osteoporosis (OP)/osteopenia (ON) and male volunteers with normal bone were recruited to this study at the Chinese PLA General Hospital (PLAGH, Beijing, China) from December 2021 to September 2023 (Table S1, Supporting Information). All these patients and volunteers involved in the study were without diabetes, thyroid diseases, autoimmune diseases, or tumors, and had not received treatment for ON or OP prior to this study. Serum samples were collected at the same time of day and under fasting conditions. CT images of the right hip from 10 elderly men with normal bone mass vs 10 elderly men with OP were included (Both groups had the equivalent baselines of age and body size, Figure S1A, Supporting Information). BMD‐based bone assessments were measured by DXA (OsteoSys EXA 3000 Bone Density Device) at the one‐third distal radius site. In men aged ≥60, OP and ON were diagnosed according to the application of the WHO diagnostic T‐score criteria to BMD measurements (with T‐scores at −1 or above defined as “normal”; between −1 and −2.5 defined as “ON”; and at −2.5 or below defined as “OP”). However, in men <60 years of age, the ethnicity or race‐adjusted Z‐scores were recommended by the International Society for Clinical Densitometry (ISCD) instead of T‐scores in the diagnostic classification (with Z‐scores of −2 or lower defined as either “low bone mineral density for chronological age” or “below the expected range for age” and those above −2 defined as “within the expected range for age”). Informed consent was obtained from all patients and normal volunteers. The study was performed with the approval of the Ethics Committee of the PLAGH.

### Animal Studies

The animal study was approved by the Ethics Committee of the Chinese Academy of Medical Sciences (No: ACUC‐A01‐2020‐014). All mice were bred and housed under specific‐pathogen‐free (SPF) conditions with a 12‐hour light/dark cycle. The wild‐type (WT) C57BL/6N mice were purchased from Beijing Vital River Laboratory Animal Technology. *Osteocalcin‐Cre* (*Ocn‐Cre*) transgenic mice express cre‐recombinase in osteoblasts were from The Jackson Laboratory (JAX:019509).

Briefly, *Ar^flox/flox^* mice were created from Cyagen Biosciences (Suzhou, China) by CRISPR/Cas‐mediated genome engineering, with two loxP sequences flanked exon 2 of mouse *Ar* gene. Then, a homozygous female *Ar^flox/flox^* mouse was bred with a male *Ocn‐Cre* mouse to generate mice that were heterozygous for the *Ar^flox/Y^* allele and heterozygous for the Cre transgene. The male mice expressing both the *Ar^flox/Y^* allele and Cre recombinase were the targeted *Ocn‐Ar^−/Y^* mice. Approximately 25% of the progeny from this mating would be the targeted mice. Twelve‐month‐old mice, maintained in a specific pathogen–free environment, were used for experiments. More details can be found in the Supporting Information.

### Tail Suspension Test

The male mice were raised to 12 weeks. Then the male mice were submitted to hind limb unloading through tail suspension or had the tail suspension device but were not suspended. Four‐week periods were performed. Each animal was single‐housed in a polycarbonate cage (25 × 18 × 20 cm) with a bearing supporting the suspension hanging system. The bearing had very low friction so that mice could easily move around on the cage surface, but they could not lean on walls with their hind limbs. The mice axis was adjusted to 30.^[^ ^74^ ^]^ Each mouse had free access to water and food.

### Cell Culture

The RAW 264.7 and MC3T3E1 cell lines were obtained from the China Infrastructure of Cell Line Resources (Beijing, China). Osteoclast formation was performed as previously described by Xiong et al.,^[^ ^75^ ^]^ with minor modifications. Briefly, to generate mature multinucleated osteoclasts, RAW 264.7 cells were cultured in α‐minimum essential medium (α‐MEM) with 10% FBS in 6‐well plates and incubated at 37 °C under 5% CO_2_. Cells were cultured by adding 10 ng mL^−1^ RANK with or without 10^−8^ m β‐E2 and testosterone to the culture system; an equal volume of DMSO was added to the control and all groups were analyzed in triplicate. The culture medium was refreshed every other day. On day 5, osteoclast cells were confirmed by TRAP staining before being harvested, and the triplicates of each group were mixed and prepared for TMT labeling. For osteoblast differentiation, MC3T3E1 cells were induced with a differentiation medium, comprising the growth medium supplemented with 50 µg mL^−1^ LAA and 10 mm β‐GP. The differentiation medium was replaced every 2 days. β‐E2 (10^−3^ m) and Tes (10^−5^ m) were dissolved in DMSO and subsequently diluted 1000‐fold in the medium for cell culture at final working concentrations 10^−6^ m for β‐E2 and 10^−8^ m for Tes. On day 15 or day 18, osteoblast cells were confirmed by Alizarin red staining before harvesting, and the triplicates of each group were mixed and prepared for TMT labeling.

### Micro‐Computed Tomography (Micro‐CT) Assessment of Bone Structure

Before fixation with 4% paraformaldehyde for 24 h, all soft tissue surrounding the femur was carefully removed. The femur bones were then securely positioned within a plastic tube to minimize any potential movement during scanning. Micro‐CT analysis was conducted utilizing a Scanco micro‐CT‐100 machine, employing the following settings: a resolution of 10 µm, X‐ray tube potential set at 70 kV, intensity at 200 µA, and an integration time of 300 ms. The raw data was processed using Scanco evaluation program v6.6 (Scanco Medical) to obtain the final results.

### Dragonfly Bone Analysis

3D reconstruction and analysis of the CT images of humans and micro‐CT images of mice were performed with the Dragonfly 2022.2 (Comet Technologies Canada, Inc.) software with DICOM files. The standardized processes were followed to determine the VOI for all bones (complete coronal position of the femoral head, and the medial part of the trochanter, and the area above the lesser trochanter of the femur). The Bone Analysis module in Dragonfly was used to segment cortical and trabecular bone^[^ ^76^, ^77^ ^]^ and to compute bone morphometric measurements, following the guidelines described by Neral M and Mologne TS et al.^[^ ^78^, ^79^ ^]^ The bone trabecular parameters were set as 0.5 mm for the proximal femur of humans and 0.1 mm for the mice. Using the Bone Analysis module, bone volume fraction (BV/TV), specific bone surface (BS/BV, mm⁻¹), and bone surface density (BS/TV, mm⁻¹) were determined for the overall description of the VOI in the proximal femur. Cortical area fraction (Ct.Ar/Tt.Ar, %), cortical porosity (Ct.Po), and cortical thickness (Ct.Th, mm) were determined for cortical bone. Trabecular thickness (Tb.Th, mm) and trabecular separation (Tb.Sp, mm) were determined for trabecular bone; trabecular number (Tb.N, mm⁻¹) was calculated based on 3D measurements for the spacing of trabeculae. In addition, the other parameters reflecting the structural and mechanical characteristics of bone tissues including endocortical perimeter (Ec.Pm, mm), periosteal perimeter (Ps.Pm, mm), structure model index, connectivity density (Conn.D, mm⁻^3^), anisotropy (MIL) and anisotropy (SVD), were determined for assessment.

### Mechanical Testing

A three‐point bending test was performed until failure of the proximal femur using an E10000 All‐Electric Dynamic Test Instrument (Instron Corporation, Canton, MA). The specimens were positioned between two supporting transverse bars (distance = 3 mm), the loading rod precisely situated at the midpoint. Utilizing a 500‐N load cell, all tests were conducted at a consistent loading rate of 3 mm min^−1^ until fracture occurred. Data acquisition was performed at intervals of 5 milliseconds. The ultimate force and stiffness were directly derived from the load and deflection curves. Elastic modulus and ultimate stress were subsequently computed using the following equations: elastic modulus = KL3/(48 Imin); ultimate stress = FultLc/(4 lmin). K = stiffness; L = Gauge‐length; I = moment of inertia; Fult = ultimate force; Lc = bone radius.^[^ ^80^ ^]^

### TRAP Staining

Mature osteoclasts were defined as TRAP‐positive cells containing three or more nuclei. TRAP staining was performed using the TRAP staining kit according to the manufacturer's instructions. Briefly, cells were washed three times with PBS and fixed with a fixative solution (65% acetone and 3.7% formaldehyde in citrate solution). Fixative solution was completely removed and cells were washed three times with ddH_2_O. Cells were stained with TRAP solution for 1 h at 37 °C in the dark.

### Alizarin Red Staining

Mineralization in osteogenic cultures was determined by Alizarin red staining. Cells were washed with PBS, fixed with 3.7% formaldehyde for 10 min, washed three times with PBS, and then stained with 40 mm Alizarin red (pH 4.1) for 15 min at room temperature. The cultures were then washed three times with ddH_2_O and photographed under a light microscope. For matrix mineralization quantification, calcium‐bound Alizarin red was solubilized with 10% acetic acid for 30 min. The supernatant was then collected and ammonium hydroxide was added to pH 4.1. The degree of mineralization was indicated by supernatant absorbance at 405 nm.

### Assay of Alkaline Phosphatase (ALP) Activity

Using an Alkaline Phosphatase Assay Kit (Beyotime, P0321), ALP activity was determined on days 3, 7, 9, 15, and 18 after MC3T3‐E1 cells were cultured in DM. The cells were trypsinized and harvested before resolution in RIPA lysis buffer. After sonication, the cell lysates were centrifuged at 12 000 rpm for 5 min, and the supernatants were added to the 96‐well plate ALP assays. All the samples and controls were assayed in triplicate. The absorbance at 405 nm was measured based on the conversion of colorless pnitrophenyl phosphate to colored p‐nitrophenol. The enzyme activity was normalized against the protein content quantified with the BCA protein assay kit.

### Bone Resorption Activity

The bone resorption capacity of osteoclasts was evaluated using the Osteo Assay Surface Plate (3987, Corning). RAW264.7 cells were seeded on the Osteo Assay Surface and stimulated with RANKL for 5 days. On day 5, cells were removed with pipetting. The remaining matrix was stained with 1% toluidine blue. The bone resorption pit image was photographed under a light microscope.

### Sample Preparation and TMT Labeling

For TMT labeling, sample preparation and labeling were performed as previously described.^[^ ^75^ ^]^ Cells were washed with cold PBS three times, followed by the addition of 8 m urea. After sonication, cell lysates were clarified by centrifugation. Supernatants were obtained and protein concentration was quantified with the Nano Drop 2000. Labeling was performed using the TMT kit following the manufacturer's protocol with slight modifications. Equal amounts of protein (100 µg) from each of the four groups were reduced with DTT and alkylated with IAA. Protein digestion was completed by incubation with Trypsin/Lys‐C at a mass ratio of 1:25 (enzyme: protein) for 12 h at 37°. The digestion was terminated by heating at 60 °C for 30 min. Digested proteins were then desalted, dried, and finally dissolved in 200 mm triethylammonium bicarbonate buffer (pH 8.5). Each group was distinguished by using different TMT labels: For osteoblast formation on day 15, TMT‐128 was used for the pre‐OB group; TMT‐129 was used for the OB group and TMT‐131 for the OB‐T group. For osteoblast formation on day 18, TMT‐127 was used for the pre‐OB group; TMT‐128 was used for the OB group, and TMT‐130 for the OB‐T group. After labeling, samples were pooled, desalted, and dissolved in 0.1% trifluoroacetic acid (TFA).

### High‐Performance Liquid Chromatography (HPLC) Separation

The TMT labeled peptides (100 µL in 0.1% TFA) were fractionated by HPLC (UltiMate 3000 UHPLC, Thermo Scientific) equipped with an Xbridge BEH300 C18 column maintained at 45 °C. The peptides were eluted by a gradient acetonitrile elution buffer (pH 10) at a flow‐rate of 1 mL min^−1^. Each 1.5 mL elution was collected as one fraction. A total of 47 fractions were collected, dried, and combined into 20 samples according to the peptide abundance. The samples were dissolved in 20 µL of 0.1% TFA for subsequent liquid chromatography (LC)‐MS/MS analysis.

### Liquid Chromatography (LC)‐MS/MS Analysis

The LC‐MS/MS analysis was performed as previously described.^[^ ^81^, ^82^ ^]^ Briefly, peptides were first passed through a homemade fused silica capillary column (75 µm ID, 150 mm length; Upchurch) packed with C‐18 resin (300 A, 5 µm; Varian, Lexington, MA, USA) column on an EASY‐nLC 1000 system using gradient TFA elution buffer (pH 1–2) at a flow‐rate of 0.3 µL min^−1^. The peptide eluate was analyzed with a directly interfaced Thermo Orbitrap Fusion mass spectrometer (Thermo Scientific) in positive‐ion mode. Xcalibur 3.0 software was used for mass data acquisition in a data‐dependent mode.

### Data Analysis

Proteins were identified using Proteome Discoverer 3.0 software (Thermo Scientific). In detail, the raw MS data files were searched against the SwissProt‐reviewed mouse proteome database. The following parameters were set as the search criteria: precursor mass tolerance, 20 ppm; fragment mass tolerance, 0.02 Da; total intensity threshold, 20 000; minimum number of peaks, 200; and a maximum of two missed cleavages were allowed. Carbamidomethylation (on C) and TMT 6‐plex (on K and the peptide N‐terminus) were set as static modifications, and oxidation (on M) was specified as a dynamic modification. Protein identification was considered valid when Score SEQUEST HT > 0 and unique peptide > 1 (if at least one peptide was statistically significant, *P *< 0.05 with a false discovery rate of 5). Reporter monoisotopic m/z was tuned according to the raw spectral data. Proteins were quantified using unique peptides. JMP software was used to analyze the distribution of protein ratios in four different groups, including log2 (OB/pre‐OB) of day 15, log2 (OB‐T/OB) of day 15, log2 (OB/pre‐OB) of day 18, and log2 (OB‐T/OB) of day 18. The differential expression cutoffs were defined as the 95% prediction interval of these four groups. Representative MS/MS spectral data of identified peptides and the intensity of TMT precursor ions were used for protein quantification.

### Bioinformatics Analysis

Gene Ontology (GO) analysis and KEGG pathway enrichment analysis were performed with DAVID (https://david.ncifcrf.gov/tools.jsp). Pathways with at least three target genes and *p* < 0.05 were considered to be statistically significant. Enriched pathways were visualized by Cytoscape (3.9.0) software. For Hierarchical Clustering analysis, the MS proteomics data presented as the ratios were input into JMP software. The STRING (Search Tool for the Retrieval of Interacting Genes/Proteins) database (http://string‐db.org) was used for predicting protein networks. All STRING network analyses were performed using UniProt accession numbers as the input and with “Experimental” and “Database” evidence at a medium (0.4) confidence level. The networks were visualized with Cytoscape to give a better idea of the potential relationships between the proteins. The networks were downloaded as tab‐delimited text files and visualized and reorganized using Cytoscape (3.9.0) software. JASPAR website (https://jaspar.elixir.no/) was used to predict the AR binding motif.

### Western Blot Analysis

For Western blot analysis, the lysed proteins (20 µg total protein) were separated by SDS‐PAGE. Gels were run according to standard methods and proteins were transferred electrophoretically to nitrocellulose membranes. Membranes were blocked with 5% non‐fat dried milk in TBS‐T (TBS plus 0.05% Tween‐20) for 30 min. The membranes were then incubated with primary antibodies overnight at 4 °C. Membranes were washed and incubated with horseradish peroxidase‐conjugated secondary antibodies and ECL reagents.^[^ ^82^ ^]^

For the Western blot of cell supernatants, the same amount of protein was loaded as the internal reference, which was validated by Ponceau S staining. In detail, the cell supernatant was collected and concentrated using a 3 kDa ultrafiltration tube. The protein concentration of the concentrated cell supernatant was determined by BCA, and a total of 25 µg protein per group was loaded for WB. After electrophoretic transfer, the membrane was stained with Ponceau S to verify that the sample loading of different groups was the same. After staining, the Ponceau S was washed off with PBS and the experiment was continued according to the WB procedure.

### Effects of Tes‐Induced Osteoblast Secretions on Osteoclastogenesis

MC3T3E1 cells were cultured in four groups (GM with DMSO, GM with 10^−8^ m testosterone, DM with DMSO, and DM with 10^−8^ m testosterone). On day 17, DMSO and testosterone were removed from the culture conditions. On day 21, culture supernatants of the osteoblasts in the four groups were collected and prepared for the subsequent assays. On day 21, osteoblasts were confirmed by Alizarin red staining. RAW 264.7 cells were cultured and supplemented with osteoblast culture supernatants; both groups were prepared in triplicate. The culture medium was refreshed every other day. On day 5, cells were confirmed by TRAP staining and photographed for calculation of the cell number and rearrangement area.

### ChIP‐qPCR

Tes‐stimulated osteoblasts were washed once in PBS and then cross‐linked with 1% formaldehyde for 15 min at room temperature. The reaction was stopped by 0.125 m glycine addition. Cross‐linked cells were then scraped for pellets and resuspended in lysis buffer (50 mm Tris⋅HCl, pH 8.1, 10 mm EDTA, 1% SDS, 1× protease inhibitor). Nuclear lysates were sonicated using a sonicator (QSONICA, Q800R, Newtown USA) to shear the DNA to a length under ≈500 bp in size. The supernatants were precleared with agarose beads for 1 h at 4 °C to and then the supernatants were incubated with antibodies against AR (Santa Cruz, sc‐7305) or isotype controls (mouse IgG, cell signaling technology, #5415). Immunoprecipitates were captured using Protein G agarose and eluted in TE buffer at 37 °C. After reverse‐crossing, the immunoprecipitated (or no IP input) DNA was analyzed via qRT‐PCR. The following qPCR primers were used for the ChIP‐qPCR analysis: region 1: F, ACTCCTATGCCCACCCTTTTAC; R, TAAACAAGGCCAGGGTTGTGGATT; region 2: F, TGAATGTCTGTGCATATGAATGGC; R, GTCACATCCTCAATTGCTTCCTT; region 3: F, TACCTGATTCCGAAGTGCATTG; R, CGCAAGAGCCTCATGACTAGAAA.

### ELISA Assay

The Mouse tenascin (TNC) ELISA Kit (CUSABIO, CSB‐EL023954MO) and Human tenascin (TNC) ELISA kit (CUSABIO, CSB‐E13125h) were used to detection of TNC in serum. The Human Amyloid Precursor Protein ELISA Kit (abcam, ab216944) and Mouse amyloid precursor protein (APP) ELISA Kit (MyBioSource, MBS2020954) were used to detect EV‐APP in serum. Mouse procollagen I N‐terminal peptide ELISA Kit (CUSABIO, CSB‐E12775m) was used to detect PINP in mouse serum. Mouse β‐CTx ELISA Kit (MyBioSource, MBS9901663) was used to detect β‐CTx in mouse serum. The ELISA assays were carried out according to the instruction manual.

### Dual Luciferase Assays

The full‐length mouse TNC promoter cDNA (−2000–+100 bp; UCSC mm39 DNA range = 63877922–63880022) was ligated into the promoter‐driven luciferase reporter plasmid pGL3‐basic. 293T cells were plated in 24‐well plates and incubated for 24 h. An expression vector encoding AR and a pRL‐TK plasmid were transiently co‐transfected with pGL3‐TNC plasmid for 48 h. A dual‐luciferase reporter assay system (Promega, Madison, WI, USA) was used to calculate luciferase activity. The Renilla luciferase gene was used as the internal reference. Relative luciferase activity = firefly luciferase activity/renilla luciferase activity.

### Molecular Docking

The Colabfold was used to predict the structures of the TNC_C terminal‐ITGαV‐ITGβ3 protein complex. The input protein sequence of TNC_C terminal was the fibrinogen C‐terminal domain (Gly1884‐Asn2099) of mouse TNC (Uniprot: Q80YX1), the extracellular segment (Phe31‐Pro983) of mouse ITGαV (Uniprot: P43406) and the extracellular segment (Asn28‐Asp717) of mouse ITGβ3 (Uniprot: O54890). The docking results were visualized using PyMOL.

### Statistical Analysis

Unless indicated otherwise, the experiments were performed in triplicate (for statistics) and repeated three times (for reproducibility). Data were presented as mean values ± SD. Statistical analysis was performed using GraphPad Prism 9 software. For two groups, the Student's *t*‐test was used. For multiple groups, ordinary one‐way analysis of variance (ANOVA) with Tukey's multiple comparisons test was used. Receiver operating characteristics (ROC) curves were computed using the Wilson/Brown method. The correlation was analyzed using Pearson's correlation coefficient. For most experiments, unless indicated otherwise, differences between groups were evaluated by Student's *t*‐test and *p *< 0.05 was considered to indicate statistical significance.

### Data Availability

The mass spectrometry proteomics data have been deposited to the ProteomeXchange Consortium (https://proteomecentral.proteomexchange.org) via the iProX partner repository^[^ ^83^, ^84^ ^]^ with the dataset identifier PXD052247. Quantitative data that support the findings of this study are available within this article and its supplementary files. All other data supporting the findings of this study are available from the corresponding authors upon reasonable request.

### Ethics Approval Statement

IRB protocol for human studies was approved by the Ethics Committee of the Chinese PLA General Hospital (No: SB2021056). All animal experiments were approved by the Ethics Committee of the Chinese Academy of Medical Sciences (No: ACUC‐A01‐2020‐014).

## Conflict of Interest

The authors declare no conflict of interest.

## Author Contributions

Y.X., M.P., and Z.Z. contributed equally to this work. W.G. and P.T. conceived and designed the experiments. Y.X., M.P., Z.Z., H.L., and X.W. performed the experiments. P.T. and W.G. contributed to the materials. Y.X., M.P., Z.Z., L.Z., W.L., and P.T. analyzed the data. Y.X., M.P., Z.Z., H.L., and X.W. contributed to the discussion. Y.X., M.P., Z.Z., and W.G. wrote the paper.

## Supporting information



Supporting Information

Supplemental Table 1

Supplemental Table 2

Supplemental Table 3

Supplemental Table 4

Supplemental Table 5

## Data Availability

The data that support the findings of this study are available from the corresponding author upon reasonable request.
