# Supplementary material for: Testosterone Delays Bone Microstructural Destruction via Osteoblast‐Androgen Receptor‐Mediated Upregulation of Tenascin‐C
Source: Adv Sci (Weinh). 2025 May 30;12(31):e01518. doi: 10.1002/advs.202501518 (PMC12376519; doi:10.1002/advs.202501518)
Supplement: Supplementary file 1 — Supporting Information [file ADVS-12-e01518-s002.docx]

**Fig. S1**


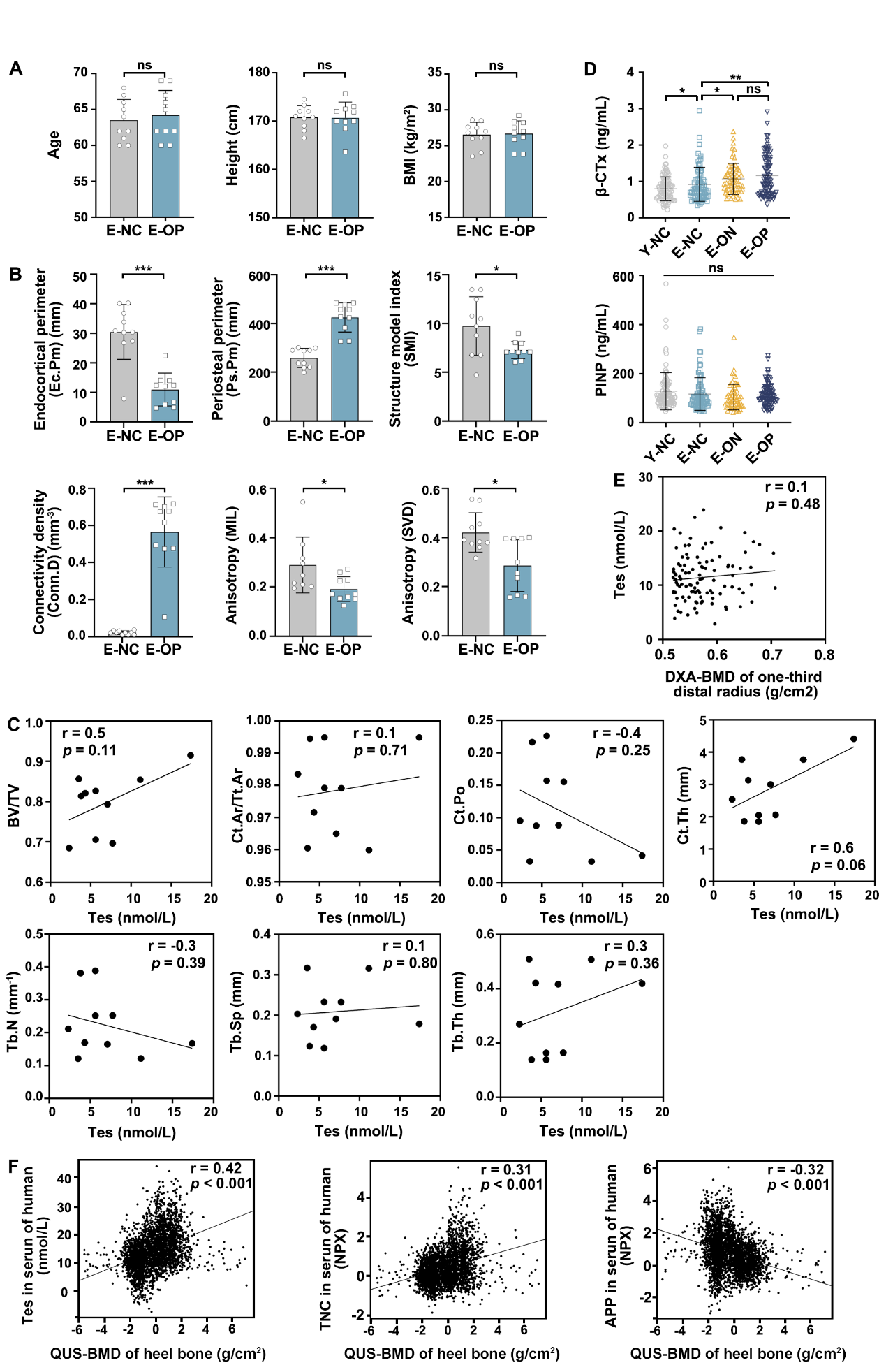


**Fig. S1 Comparisons of physical characteristics and bone analysis between elderly men with normal BMD and OP.** (A) Comparisons of the age, height, and BMI between elderly men with normal BMD and OP. n = 10 per group. (B) Bone analysis from CT images of right hip of elderly men; n = 10 per group. (C) Spearman’s correlation analysis between Tes level and parameters of bone analysis. (D) The measurement of biochemical markers of bone turnover. (E) Spearman’s correlation analysis between serum Tes level and DXA-BMD of one-third distal radius in young men (n = 105). (F) Spearman’s correlation analysis between serum Tes, TNC, APP level and QUS-BMD of heel bone in elderly men over 60 years of age from the UK Biobank (n = 3097). Student's *t* test was used for two groups comparisons, one‐way ANOVA with Tukey’s multiple comparisons test was used for multiple comparisons. All tests were two‐sided; **p* < 0.05; ***p* < 0.01; ****p* < 0.001; ns = no significance. BMI = body mass index; E-NC = elderly men with normal BMD; E-OP = elderly men with osteoporosis; DXA = dualenergy X-ray absorptiometry; Tes = testosterone; BMD = bone mineral density; QUS = quantitative ultrasound.

**Fig. S2**

**
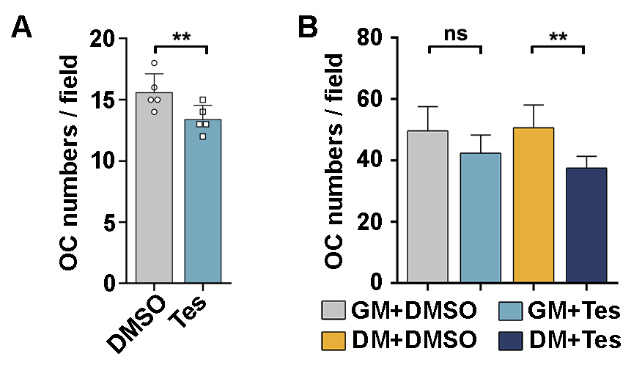
**

**Fig. S2 Tes inhibits osteoclast formation.** (A) Quantification of TRAP^+^ osteoclasts treated with Tes, compared with controls (DMSO). (B) Quantification of TRAP^+^ osteoclasts treated with GM or DM supernatant derived from MC3T3E1. All results are representative of data from at least three independent experiments. Student's *t* test was used for two groups comparisons, one‐way ANOVA with Tukey’s multiple comparisons test was used for multiple comparisons. All tests were two‐sided; ***p* < 0.01, ns = no significance. OC = osteoclast; GM = general medium derived from MC3T3E1 culture supernatant; DM = differentiation medium derived from MC3T3E1 culture supernatant.

**Fig. S3**


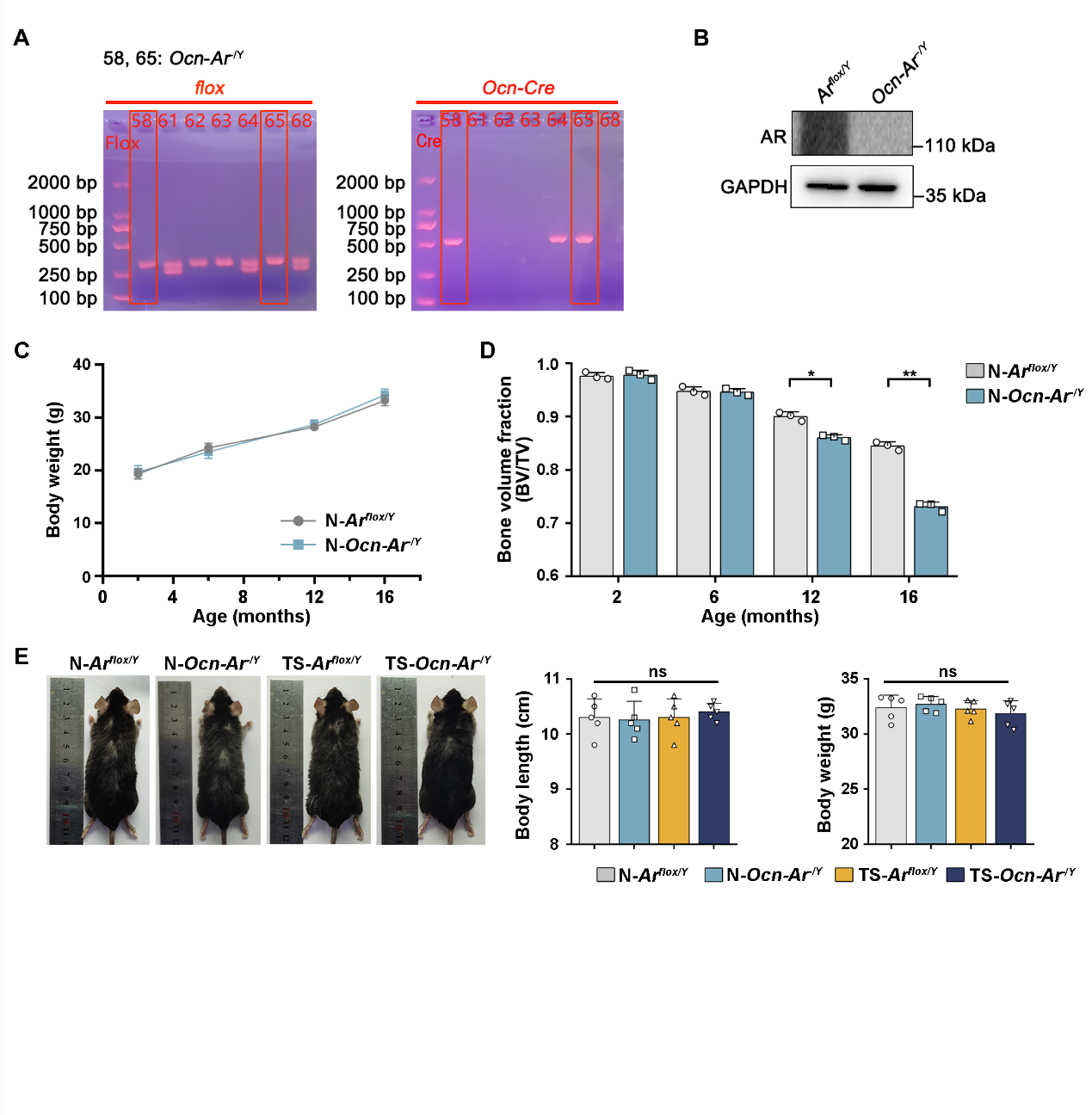


**Fig. S3 Comparisons of physical characteristics and bone analysis between *Ar^flox/Y^* and *Ocn-Ar^−/Y^* mice.** Validation of *Ar* knockout specifically in osteoblasts by PCR (A) and western blot (B). (C) Changes in body weight of *Ar^flox/Y^* and *Ocn-Ar^−/Y^* male mice at the age of 2, 6, 12 and 16 months old. n = 3 per group. (D) Comparisons of BV/TV between *Ar^flox/Y^* and *Ocn-Ar^−/Y^* male mice at the age of 2, 6, 12 and 16 months old. n = 3 per group. (E) Comparisons of body length and weight of *Ar^flox/Y^* and *Ocn-Ar^−/Y^* mice (male, 14-month-old) with or without tail suspension; n = 5 per group. Student's *t* test was used for two groups comparisons, one‐way ANOVA with Tukey’s multiple comparisons test was used for multiple comparisons. All tests were two‐sided; **p* < 0.05; ***p* < 0.01; ns = no significance. N = without tail suspension; TS = with tail suspension.

**Fig. S4**


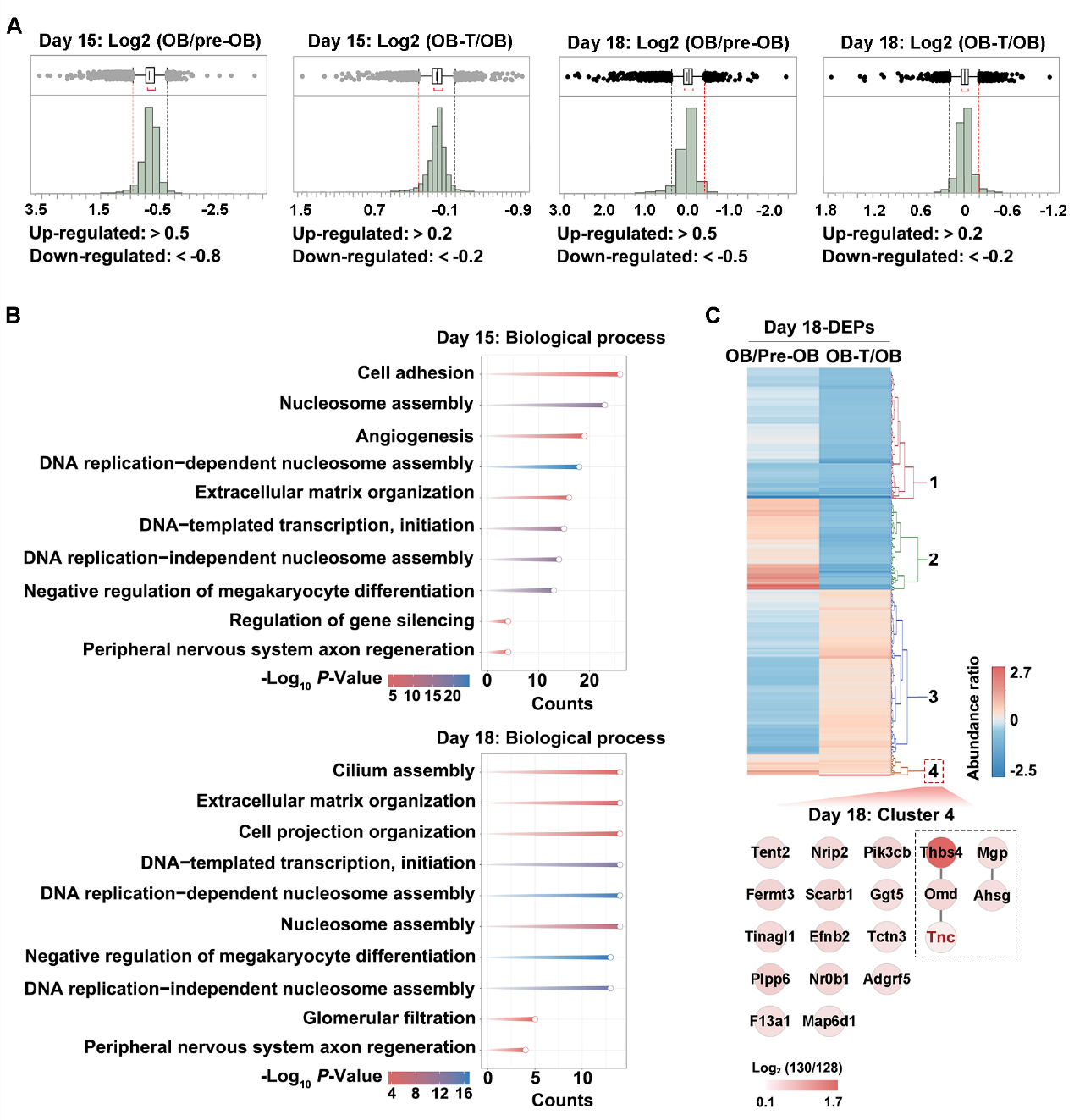


**Fig. S4** **Bioinformatic analysis of DEPs in the process of osteoblast differentiation and Tes-stimulated osteoblast differentiation.** (A) The cut-off ratio of DEPs. (B) Biological process analysis of DEPs at day 15 and day 18 in the process of osteoblast differentiation and Tes-stimulated osteoblast differentiation. (C) Hierarchical clustering heatmaps of DEPs at day 18 in the process of osteoblast differentiation and Tes-stimulated osteoblast differentiation (upper) and STRING analysis of the DEPs in the Cluster 4 (bottom).

**Fig. S5**


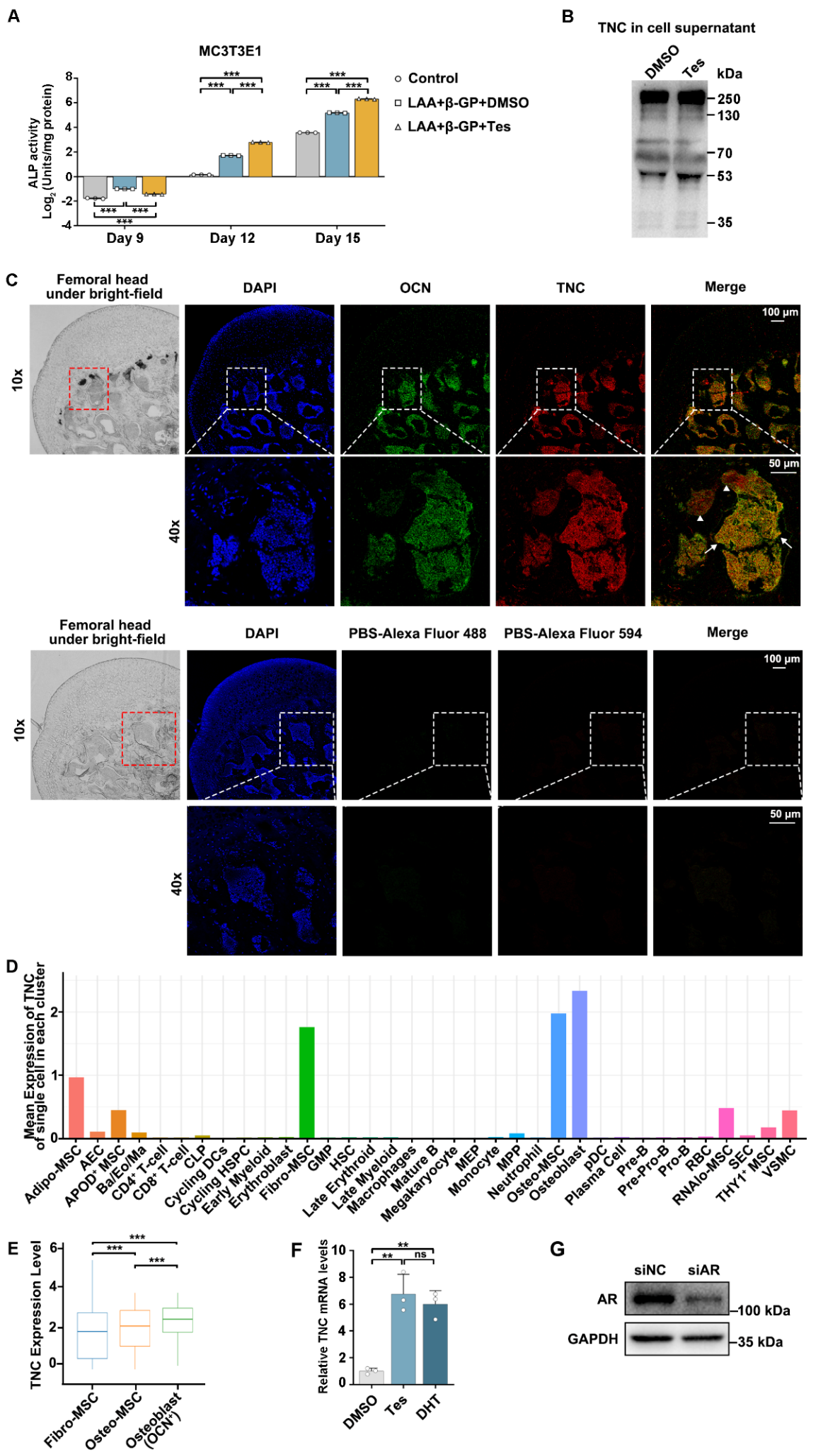


**Fig. S5 ALP activity, primary source of TNC and verification of AR knockdown.** (A) ALP activity of MC3T3E1 on days 9, 12, and 15 in the process of osteogenic differentiation. (B) The result of Western blot whole membrane. (C) Immunofluorescence co-staining reveals colocalization of TNC with OCN^+^ osteoblasts in mouse femoral head. (D) Histograms of mean expression of TNC of single cell in 35 cell clusters. (E) Box plots of single-cell TNC expression in the three highest-expression clusters. (F) Relative TNC mRNA levels in Tes or DHT stimulated osteoblasts. (G) Verification of AR-knockdown in osteoblasts by Western blot. Student's *t* test was used for two groups comparisons, one‐way ANOVA with Tukey’s multiple comparisons test was used for multiple comparisons, Wilcoxon non-parametric test was used to analyze the TNC expression levels of single cell in the three highest-expressing groups (Osteoblast, Osteo-MSC and Fibro-MSC). All tests were two‐sided; ***p* < 0.01; ****p* < 0.001; ns = no significance. ALP = alkaline phosphatase; β-GP = β-glycerophosphate; LAA = L-ascorbic acid; Tes = testosterone.

**Fig. S6**


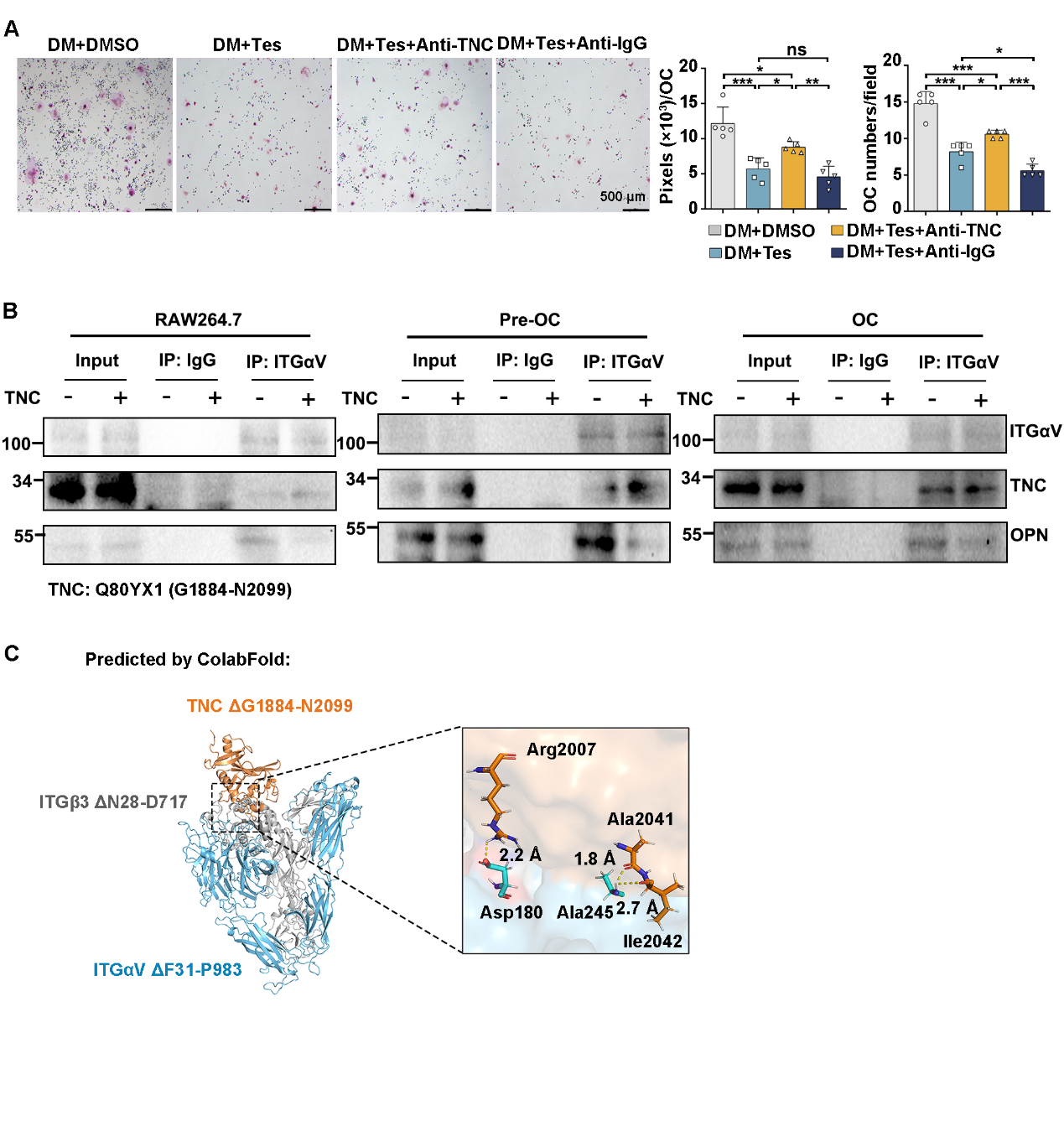


**Fig. S6 The fibronectin C-terminus of TNC inhibits the differentiation of osteoclasts and binding sites were predicted using ColabFold docking analysis.** (A) Representative images and quantification of TRAP staining. Data were analyzed using one-way ANOVA. **p* < 0.05; ***p* < 0.01; ****p* < 0.001; ns = no significance. Tes = testosterone; DM = differentiation medium derived from MC3T3E1 culture supernatant. (B) RAW264.7, Pre-OC (40h RANKL-induced RAW264.7 cells), and OC cell lysates were immunoprecipitated with anti-ITGαV antibody or control IgG. Western blot analysis was performed to detect co-precipitated proteins. (C) Overview of ColabFold docking results for TNC-C (Gly1884-Asn2099, orange) with integrins αV (Phe31-Pro983, blue) and β3 (Asn28-Asp717, grey).

**Fig. S7**

**
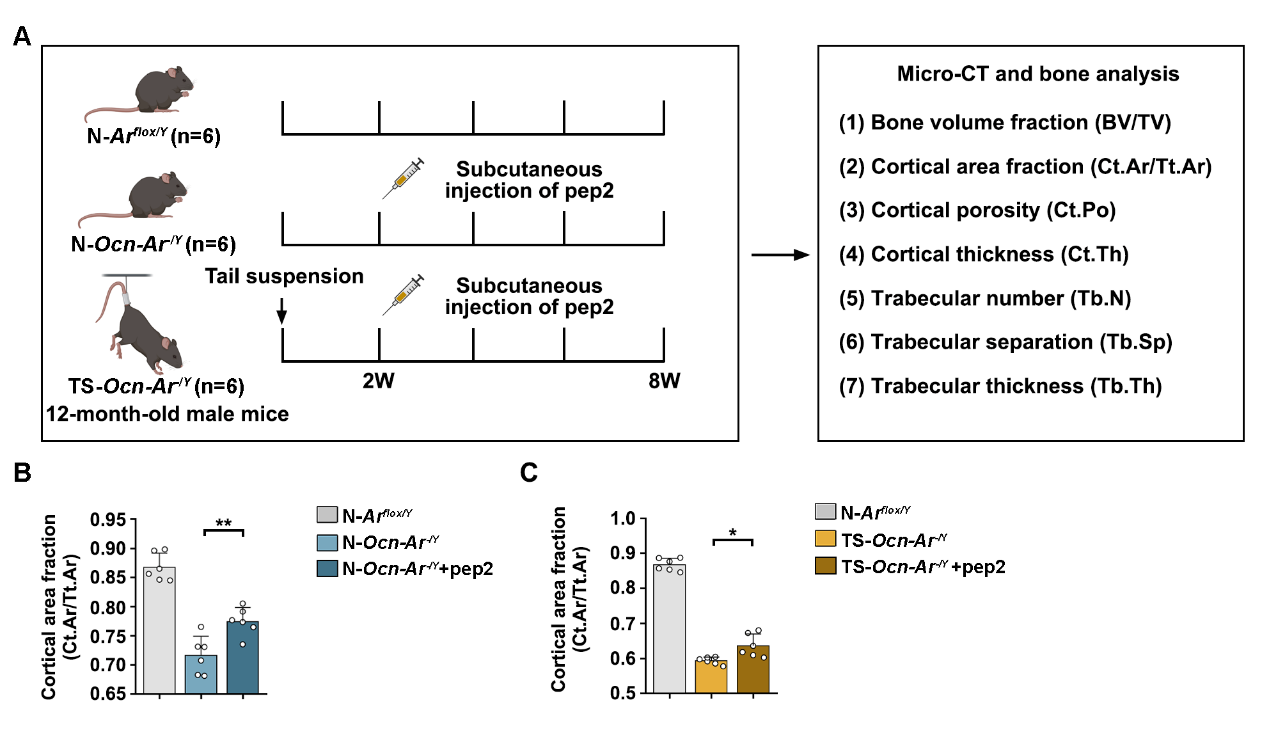
**

**Fig. S7 Construction of male elderly male elderly osteoporotic mice and bone analysis.** (A) Experimental flow diagram. (B-C) Cortical area fraction analysis of micro-CT images of femur of *Ocn-Ar^−/Y^* mice (male, 14-month-old) with or without tail suspension to determine the therapeutic effect of TNC peptides. One‐way ANOVA with Tukey’s multiple comparisons test was used for multiple comparisons. All tests were two‐sided; **p* < 0.05; ***p* < 0.01.

**Fig. S8**

**
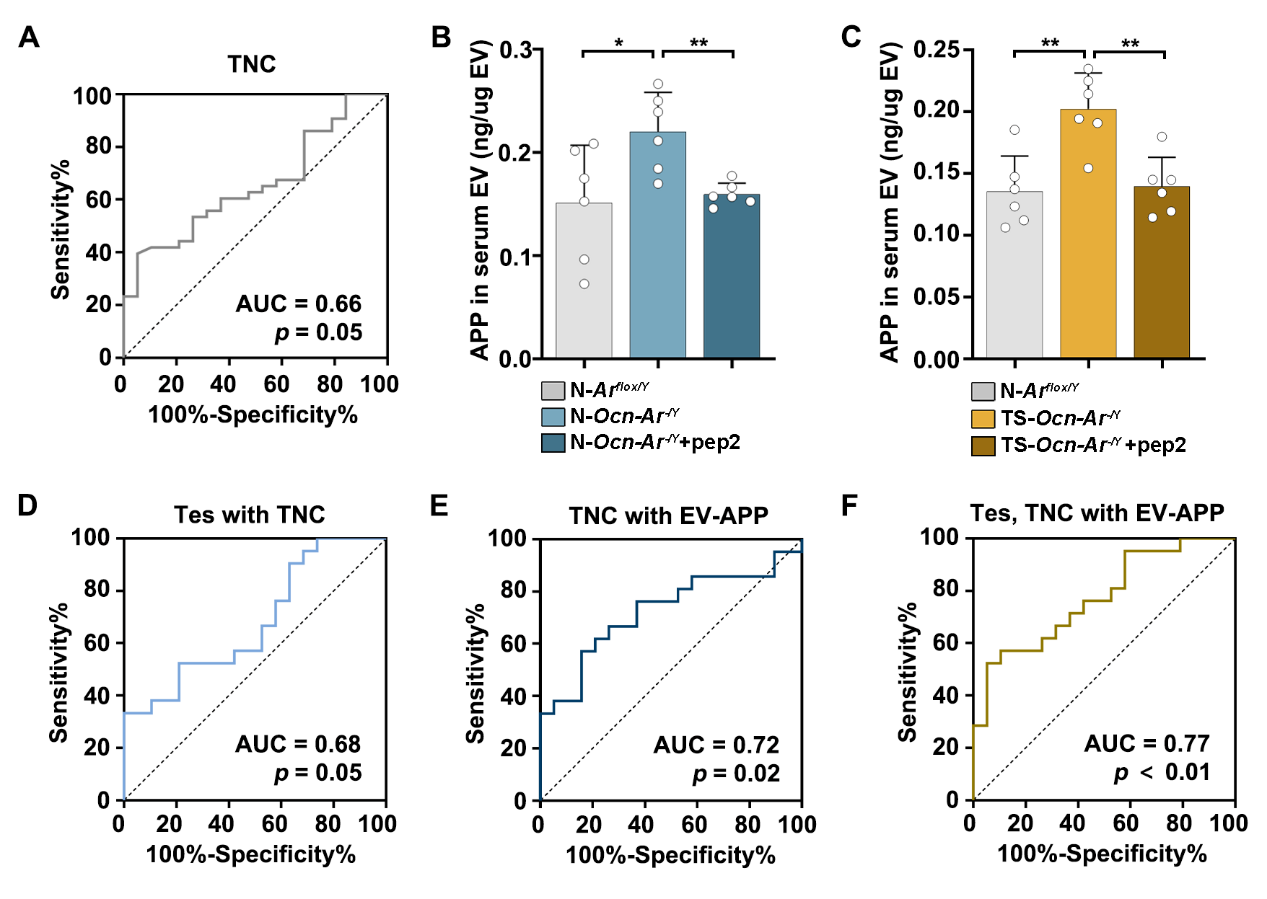
**

**Fig. S8 The clinical significance of serum EV-APP.** (A) ROC curve analysis of serum TNC. (B-C) APP in serum EV of osteoblast-specific AR knockout mice after TNC-C pep2 treatment detected by ELISA. (D-F) ROC curve analysis of combined markers. AUC = area under curve. One‐way ANOVA with Tukey’s multiple comparisons test was used for multiple comparisons. All tests were two‐sided; **p* < 0.05; ***p* < 0.01.

**Fig. S9**


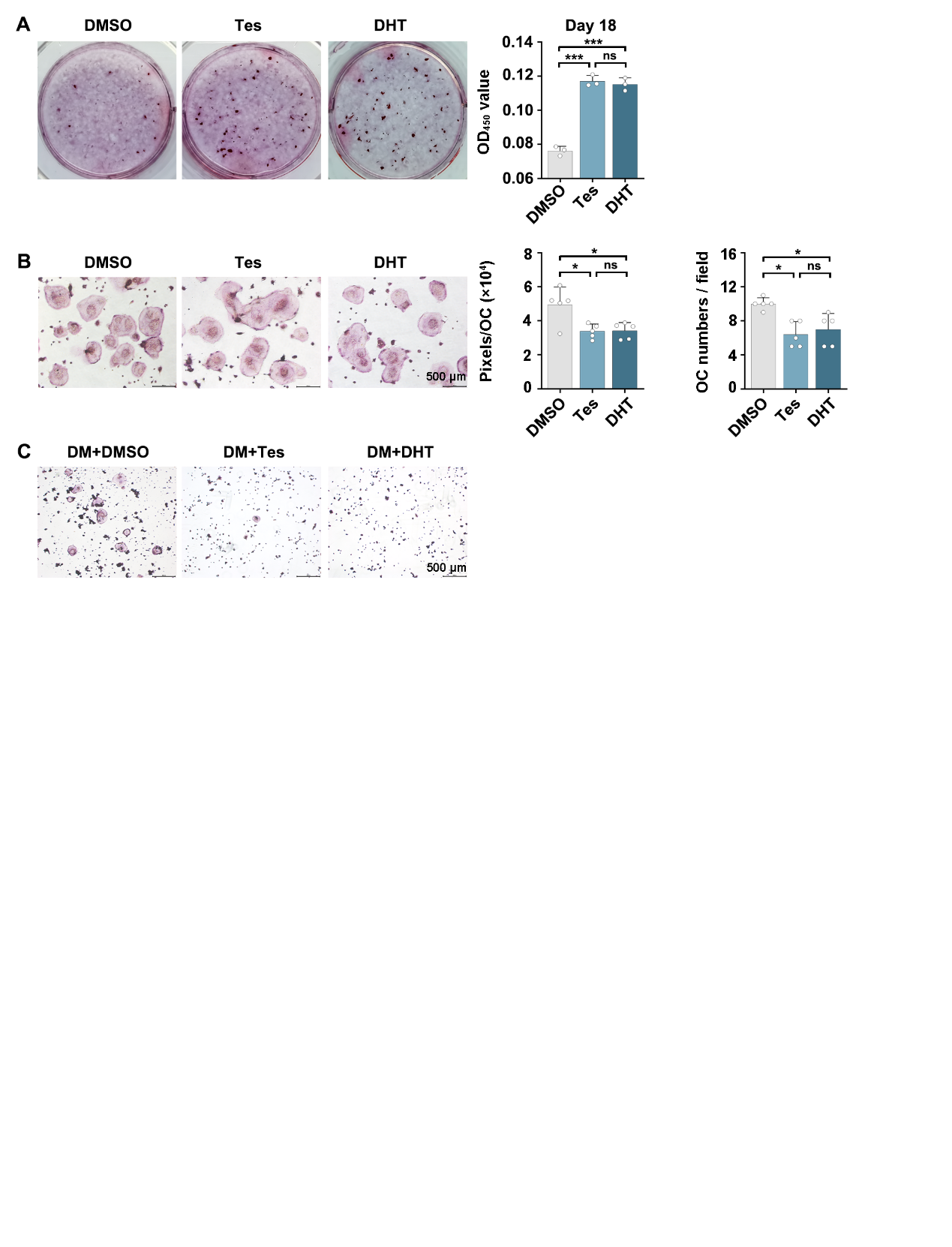


**Fig. S9** **The concentrations of 10^-8^ M Tes and 10^-9^ M DHT had similar effects.** (A) Representative images and quantification of Alizarin Red staining of MC3T3E1 (osteoblast precursor) cells after 18 days of osteogenic differentiation in the presence of Tes or DHT. (B) Representative images and quantification of TRAP staining of osteoclasts treated with Tes or DHT compared with controls (DMSO). (C) Representative images of TRAP staining of osteoclasts treated with MC3T3E1-derived DM supernatant. Student's *t* test was used for two groups comparisons, one‐way ANOVA with Tukey’s multiple comparisons test was used for multiple comparisons. All tests were two‐sided; **p* < 0.05; ****p* < 0.001; ns = no significance. Tes= testosterone; DHT= dihydrotestosterone.

**Fig. S10**

**
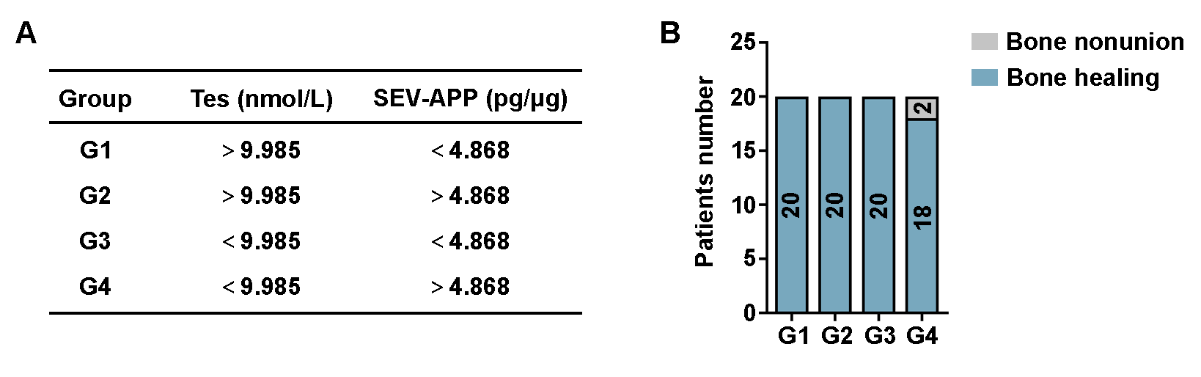
**

**Fig. S10 The potential clinical significance of serum Tes combined with SEV-APP in elderly OP men.** (A) The information of the groups. (B) The numbers of bone nonunion patients in each group. Tes = testosterone.

**Supporting Experimental Section**

**Animal studies**

***Ar^flox/flox^* mice**.

*Ar^flox/flox^* mice were generated by Cyagen Biosciences (Suzhou, China) using CRISPR/Cas-mediated genome engineering. Two loxP sites were inserted to flank exon 2 of the mouse *Ar* gene. Genotyping was performed by PCR to identify homozygous, heterozygous, and wild-type mice using PCR primer 1.

PCR primers 1 for insertion of loxP:

Forward primer (F1) 5’-AAGGGACACTGAGAGACTCAAGAAG-3’.

Reverse primer (R1) 5’-CTTCTTCCAGAGAGAGGCCTATGA-3’.

Expected size: WT (no insertion of loxP), 269 bp; MT (insertion of loxP), 319 bp.

Homozygous: one band with 319 bp,

Heterozygous: two bands with 319 bp and 269 bp,

WT: one band with 269 bp.

***Ocn-Cre* mice**.

Ocn-Cre transgenic mice, widely used for osteoblast-specific gene targeting, were purchased from The Jackson Laboratory (JAX:019509). Genotyping of the Cre transgene was performed using PCR with specific primers.

PCR primers Cre for Cre transgene:
Forward primer (Cre-F): 5’-GAACGCACTGATTTCGACCA-3’
Reverse primer (Cre-R): 5’-GCTAACCAGCGTTTTCGTTC-3’
Expected amplicon size: 204 bp

***Ocn-Ar^-/Y^* mice generation**.

Given that the *Ar* gene is located on the X chromosome, male mice with osteoblast-conditional knockout of *Ar* were generated by breeding female *Ar^flox/flox^* mice (C57BL/6N background) with male *Ocn-Cre* transgenic mice. Male offspring carrying both the *Ar^flox/Y^* allele and Cre transgene were identified as the targeted Ocn*-Ar^-/Y^* mice. Genotyping was performed using DNA extracted from toe tissue, with PCR primers Cre specific for Cre transgene (expected size: 204 bp) and PCR primers 1 specific for loxP site insertion (expected size: one single 319 bp band for the mutant allele). Approximately 25% of the offsprings from this mating were the desired genotype. Twelve-month-old targeted mice were used for subsequent experiments. Primary osteoblasts were isolated from these mice, and osteoblast-specific *Ar* knockout was confirmed by western blot analysis.

**Serum samples and computed tomography (CT) images of recruited individuals**

**Methods for obtaining Serum samples**

All patients gave informed consent before obtaining the samples, the median elbow vein was selected and skin was sterilized, venipuncture was performed using aseptic technique, blood was collected into blood collection tubes containing separator gel, gently inverted and mixed, the blood was allowed to stand and stratify and then centrifuged to separate the serum, the serum was carefully transferred to sterile containers, the samples were labeled and stored.

**Methods for performing computed tomography scanning**

All patients gave informed consent before CT scanning was performed, the CT scanning procedure is as follows: the examinee needs to remove metal jewelry or objects that may affect the X-ray penetration of the examined area before the examination, take the supine position, rotate the toes of both feet to the medial side and bring them together, raise the upper arms of both feet, lay the body flat and straight, and the head is advanced, the examinee maintains the position of the body unmoving during the scanning process, and the scope of the scanning is scanned from the top of the acetabulum by 1 to 2 cm downward continuously, including the hip joints on both sides.

**UK Biobank Data Acquisition and Analysis**

UK Biobank (https://www.ukbiobank.ac.uk/) is a large-scale biomedical database containing detailed genetic, health, and phenotypic information from 502,364 participants aged 40–69 years at recruitment.

A total of 3,097 elderly men over 60 years of age with complete records on demographics (age, race, income, education, BMI), laboratory tests (serum testosterone, oestrogen, calcium, phosphate), lifestyle factors (smoking, alcohol consumption), proteomic markers (TNC, APP), and quantitative ultrasound (QUS)-measured BMD of heel bone were included. We conducted multivariable linear regression analyses to assess the correlations between serum testosterone, TNC, APP, and BMD, adjusting for relevant covariates. Pearson correlation coefficients (r) and p-values were calculated, with statistical significance defined as *p* < 0.05. All analyses were performed using R software (version 4.4.1).

**Section Preparation and Immunofluorescence Double-Staining**

Freshly dissected mouse femur was embedded in optimal cutting temperature (OCT) compound and snap-frozen in liquid nitrogen. After sectioning, the slides were washed three times in PBS (5 min per wash) to remove residual OCT, followed by permeabilization with 0.3% Triton X-100 in PBS for 10 min at room temperature (RT), and blocked with 5% bovine serum albumin (BSA) for 1 h at RT. For double-staining, sections were incubated overnight at 4°C with primary antibodies: anti-OCN (1:250, Servicebio, GB11233) and anti-TNC (1:250, Proteintech, 67710-1-Ig). After washing with PBS, sections were probed with Goat anti-Rabbit IgG (H+L) Highly Cross-Adsorbed Secondary Antibody Alexa Fluor™ 488 (1:250, Thermo, A-11034) and Goat anti-Mouse IgG (H+L) Highly Cross-Adsorbed Secondary Antibody Alexa Fluor™ 594 (1:250, Thermo, A-11032) for 1 h at RT. Nuclei were counterstained with DAPI (5 μg/mL) for 5 min. Images were acquired using a confocal microscope (Leica Laser Scanning Confocal Microscopy SP8 STED 3X) with consistent exposure settings across samples. Negative controls omitting primary antibodies confirmed staining specificity.
